# Tandem mass tag-based (TMT) quantitative proteomics analysis reveals the response of fine roots to drought stress in cotton (*Gossypium hirsutum* L.)

**DOI:** 10.1186/s12870-020-02531-z

**Published:** 2020-07-11

**Authors:** Shuang Xiao, Liantao Liu, Yongjiang Zhang, Hongchun Sun, Ke Zhang, Zhiying Bai, Hezhong Dong, Yuchun Liu, Cundong Li

**Affiliations:** 1 College of Agronomy, Hebei Agricultural University/ State Key Laboratory of North China Crop Improvement and Regulation / Key Laboratory of Crop Growth Regulation of HeBei Province, Baoding, 071001 Hebei China; 2 Cotton Research Center/ Key Laboratory of Cotton Breeding and Cultivation in Huang-huai-hai Plain, Ministry of Agriculture, Shandong Academy of Agricultural Sciences, Jinan, 250100 Shandong China

**Keywords:** Cotton, Fine roots, TMT, Proteome, Drought stress

## Abstract

**Background:**

Cotton (*Gossypium hirsutum* L.) is one of the most important cash crops worldwide. Fine roots are the central part of the root system that contributes to plant water and nutrient uptake. However, the mechanisms underlying the response of cotton fine roots to soil drought remains unclear. To elucidate the proteomic changes in fine roots of cotton plants under drought stress, 70–75% and 40–45% soil relative water content treatments were imposed on control (CK) and drought stress (DS) groups, respectively. Then, tandem mass tags (TMT) technology was used to determine the proteome profiles of fine root tissue samples.

**Results:**

Drought significantly decreased the value of average root diameter of cotton seedlings, whereas the total root length and the activities of antioxidases were increased. To study the molecular mechanisms underlying drought response further, the proteome differences between tissues under CK and DS treatments were compared pairwise at 0, 30, and 45 DAD (days after drought stress). In total, 118 differentially expressed proteins (DEPs) were up-regulated and 105 were down-regulated in the ‘DS30 versus CK30’ comparison; 662 DEPs were up-regulated, and 611 were down-regulated in the ‘DS45 versus CK45’ comparison. The functions of these DEPs were classified according to their pathways. Under early stage drought (30 DAD), some DEPs involved in the ‘Cutin, suberin, and wax synthesis’ pathway were up-regulated, while the down-regulated DEPs were mainly enriched within the ‘Monoterpenoid biosynthesis’ pathway. Forty-five days of soil drought had a greater impact on DEPs involved in metabolism. Many proteins involving ‘Carbohydrate metabolism,’ ‘Energy metabolism,’ ‘Fatty acid metabolism,’ ‘Amino acid metabolism,’ and ‘Secondary metabolite biosynthesis’ were identified as DEPs. Additionally, proteins related to ion transport, stress/defense, and phytohormones were also shown to play roles in determining the fine root growth of cotton plants under drought stress.

**Conclusions:**

Our study identified potential biological pathways and drought-responsive proteins related to stress/defense responses and plant hormone metabolism under drought stress. Collectively, our results provide new insights for further improving drought tolerance in cotton and other crops.

## Background

Drought is an important abiotic stress factor that severely limits the growth and development of cotton plants and reduces cotton yields [1, 2]. Among the various plant organs, the root system first perceives drought signals, which leads to a series of plant drought responses at the morphological, physiological, and cellular levels [3]. Fine roots (i.e., roots with diameters of less than 2 mm) act as the major constituents of root systems and play a leading role in regulating the total length and surface area of whole root systems [4]. They are the most active determinant of the physiological functions of root systems and control the uptake of water and nutrients [5]. In addition to genetic background, environmental factors significantly impact the architecture of fine roots [6]. Soil water status is a critical environmental factor affecting the growth and distribution of fine roots, with a response to drought that is largely determined by the physiological adaptability of plants to water deficiency [7]. Thus, understanding the response of fine roots to soil water deficiency in order to elucidate plant drought resistance mechanisms is of considerable scientific merit.

Understanding the mechanisms of plant drought response is also important for developing strategies to improve crop drought tolerance. At the molecular level, plant responses to environmental stresses can be accomplished by changing the expression of stress-associated genes [8]. However, biological processes cannot be explained directly by the transcript levels and translation efficiencies of genes because estimates of these processes only roughly estimate protein expression levels [9]. Additionally, many proteins undergo further post-translational modifications, which act with expression to determine the stress-associated responses of plants [10].

At present, proteomic analyses have strengthened our understanding of the mechanisms of physiological adaptations to stress in plants. Many researchers have investigated the proteomic response of cotton plants under drought stress. For example, Zheng et al. [11] used two-dimensional electrophoresis to investigate the molecular mechanisms of cotton fiber elongation in response to drought stress and identified 132 differentially expressed proteins. Zhang et al. [12] comparatively analyzed leaf proteomes of drought-responsive proteins in drought-tolerant and drought-sensitive cotton cultivars, and the identified proteins were mainly involved in metabolism, antioxidants, transport, and cellular structure. Lu et al. [13] found that the differentially expressed proteins in cotton leaves under drought stress were mainly involved in ‘Photosynthesis,’ ‘Material and energy metabolism,’ ‘Material transport,’ and ‘Stress defense.’ In these studies, many proteins were found to be involved in the metabolic pathways and cellular processes that are likely related to cotton drought resistance. However, at the proteomic level, there is little information about the fine root response of cotton plants under drought stress.

The recent development of a high-sensitivity proteomic platform based on isobaric labels tandem mass tags (TMT) has provided a powerful approach to comprehensively and accurately analyzing low-abundance proteins [14]. Thus, we employed a quantitative proteomic analysis based on TMT labels, coupled with liquid chromatography-tandem mass spectrometry (LC-MS/MS), to capture the differential protein expression profiles between the fine roots of cotton plants subjected to two irrigation treatments (70–75% and 40–45% soil relative water content). The main objectives were as follows: (i) evaluation of proteomic changes in the fine roots of plants under drought stress; (ii) elucidation of the crucial metabolic pathways responsible for the establishment of fine roots across two critical drought stages, 30 and 45 days after drought stress (DAD). The results obtained from the present study help to clarify the molecular mechanisms of drought stress responses in cotton plants.

## Results

### Morphological responses of aboveground tissues to drought stress

**Fig. 1:**
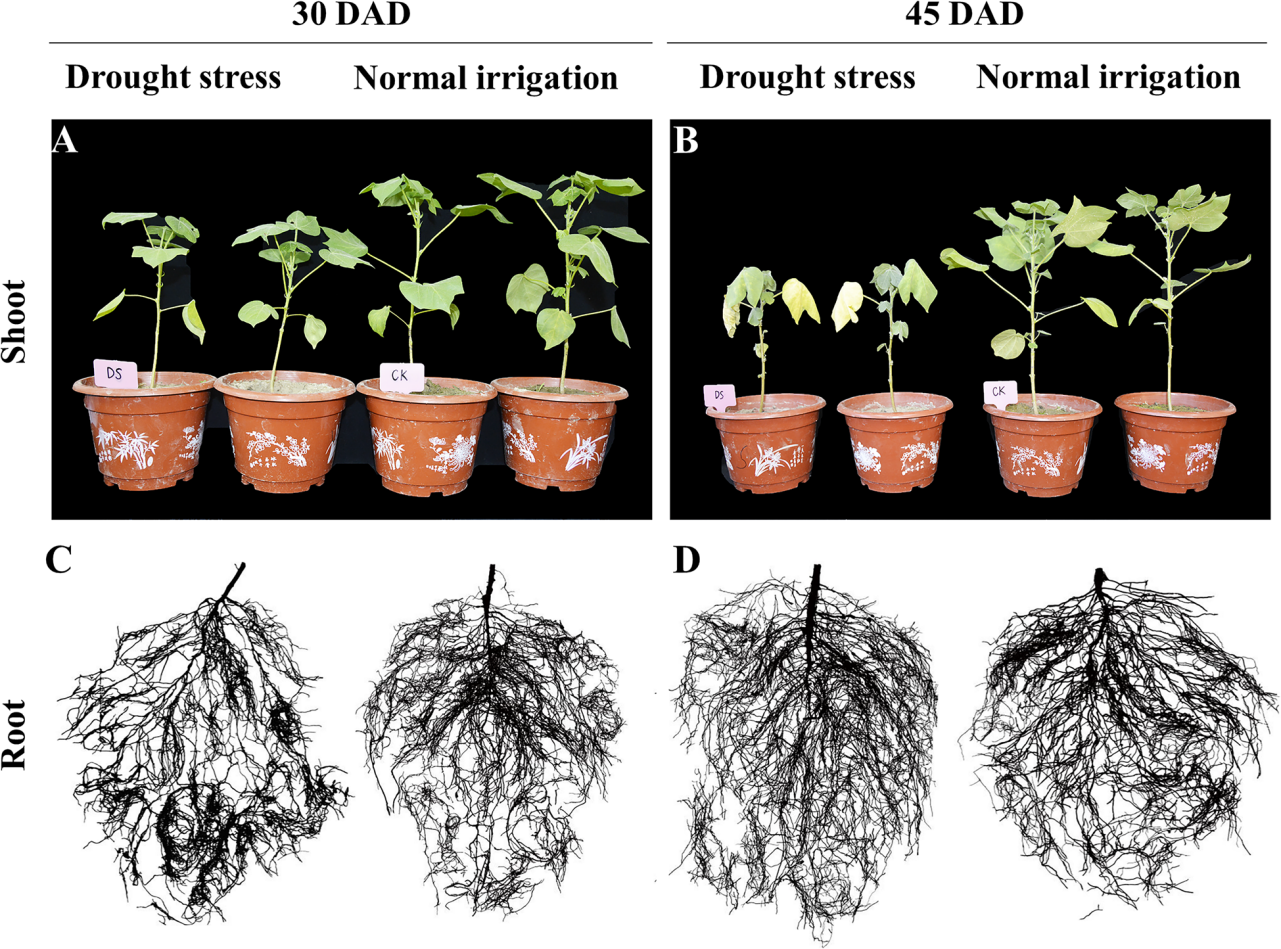
Effects of drought stress on the morphology of shoots and roots of cotton plants. Shoot morphology at 30 days after drought (DAD) (**a**). Root morphology at 30 DAD (**c**). Plant shoot morphology at 45 DAD (**b**). Root morphology at 45 DAD (**d**)

To confirm how drought stress influenced the development of cotton plants, we first surveyed the morphological traits of plants at five time points (i.e., 0, 15, 30, 45, and 60 DAD). As expected, long periods of drought stress negatively affected the aboveground portions of plants (Fig. 1a, b). Under drought stress, plant height and stem diameter showed a significant decrease at 15 DAD compared with the CK treatment (*P* < 0.05), with a much greater reduction by 30 DAD (*P* < 0.01) (Additional file 1: Fig. S1). The leaves of CK plants remained greenish across various timepoints, and there was no difference in leaf area between DS and CK treatments at 15 DAD. Up to 30 DAD, the leaves of the DS group were wilted to some extent (Fig. 1a), while leaf area and leaf thickness began to decrease (*P* < 0.05). Leaf wilting intensified by 45 DAD, with a large amount of shedding occurring among the lower leaves, while the remaining leaves of the plants were yellowish (Fig. 1b).

### Morphological responses of roots to drought stress

Root morphology changed throughout the soil drought stress treatment (Fig. 1c, d; Additional file 2: Fig. S2). Reflecting results based on plant shoots, both root length and root average diameter were similar under CK and DS treatments at 15 DAD. At 30 DAD, the root length of DS plants increased by 16.5% compared with the CK treatment. In contrast, mean root diameter was decreased by drought, with a 5.0% reduction of DS plants compared to CK plants. Moreover, the effects of drought on this trait increased as the treatment progressed. The difference in root length behavior between CK and DS treatments was most pronounced at 45 DAD, with a 20.9% increase of DS plants compared with CK plants. Average diameter under the CK treatment gradually increased with root system development, while that under DS was constant (i.e., 0.31–0.33 mm). Root length and mean diameter determines the surface area and projected area of the root system, but the growth trends of the above root traits were similar between CK and DS treatments during the experiment.

### Effects of drought stress on physiological performance

**Fig. 2:**
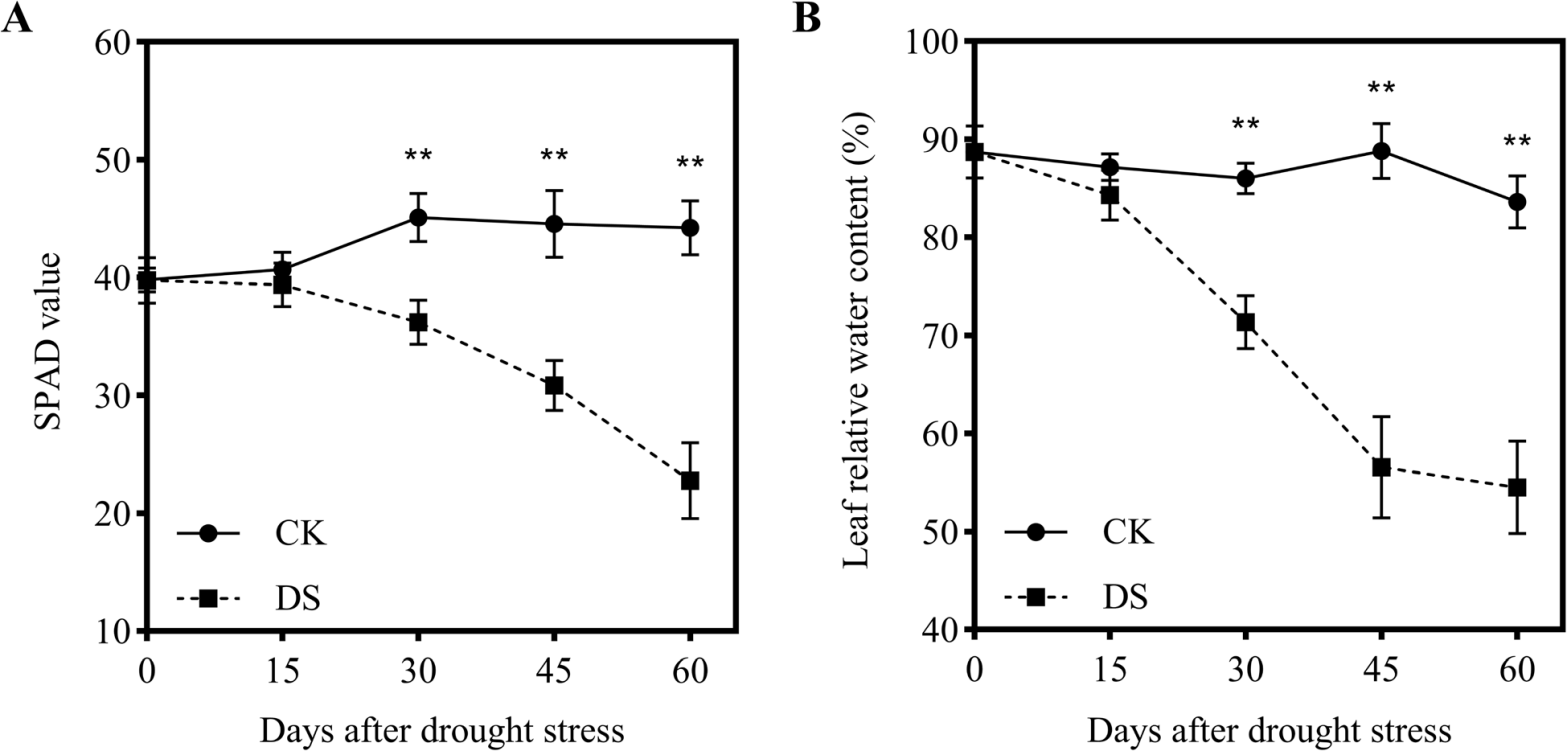
Changes in soil and plant analyzer development (SPAD) values (**a**) and leaf relative water content (LRWC) (**b**) of plants under drought stress. Each data point represents the mean of five independent biological replicates (mean ± SD). Asterisks indicate statistically significant differences compared with the control (^*^P < 0.05; ^**^P < 0.01)

Soil and plant analyzer development (SPAD) readings can predict the chlorophyll content of crops. In this study, the SPAD values of CK plants remained stable from 0 to 60 DAD. However, SPAD values gradually decreased with DS progression, first showing a significant difference at 30 DAD (*P* < 0.01) compared with 0 DAD. At 15, 30, 45, and 60 DAD, SPAD values decreased by 3.2, 19.7, 30.8, and 48.6% relative to 0 DAD, respectively (Fig. 2a). Leaf relative water content (LRWC) is another important physiological indicator for evaluating plant responses to drought stress. Similar to SPAD, the LRWC of leaves remained stable (83–89%) under the CK treatment and sharply declined by 30 and 45 DAD under DS, being 17.0 and 36.3% lower than that under CK conditions, respectively (Fig. 2b).

### Responses of antioxidant enzymes to drought stress

**Fig. 3:**
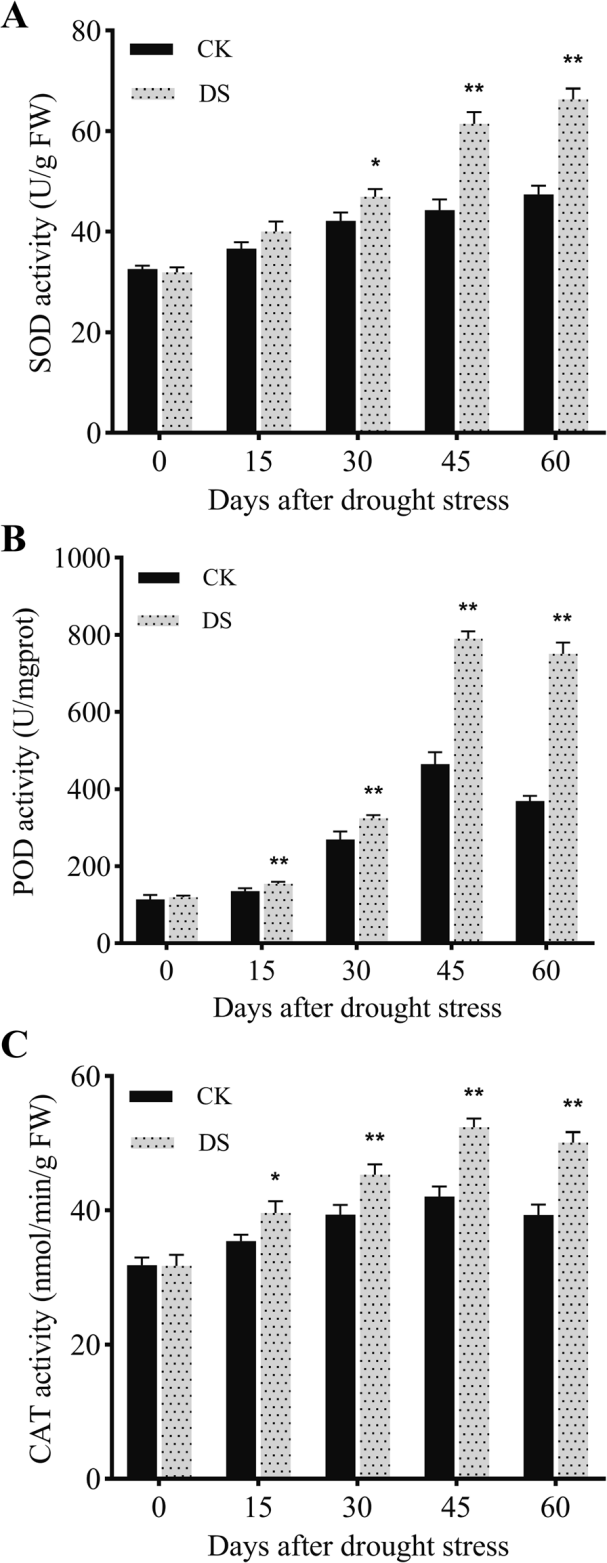
Changes in superoxide dismutase (SOD) **(a)**, peroxidase (POD) **(b)**, and catalase (CAT) activities **(c)** of fine roots under normal irrigation (CK) and drought (DS) treatments at different time points. Each data point represents the mean of five independent biological replicates (mean ± SD). Asterisks indicate statistically significant differences compared with the control (^*^P < 0.05; ^**^P < 0.01)

The activities of reactive oxygen species (ROS) scavenging enzymes in response to drought stress are shown in Fig. 3. The activities of superoxide dismutase (SOD) were significantly different (*P* < 0.05) at 30 DAD, and the peroxidase (POD) activities showed a difference at 15 DAD (*P* < 0.01), which indicated that POD activity was more sensitive to drought stress than was SOD. The activities of catalase (CAT) differed at 15 DAD (*P* < 0.05) and were enhanced by drought stress (Fig. 3c). Compared with CK plants, the CAT activities under DS increased significantly by 15 DAD, increased further by 30 DAD, and reached their peak at 45 DAD.

### Overview of quantitative proteomic responses to drought stress

A total of 11,628 proteins were identified, of which 10,344 contained quantitative information. The length distribution analysis of peptides showed that most of them consisted of 7–20 amino acids, which is in accordance with the quality control requirements (Additional file 3: Fig. S3A). A further analysis was performed to test whether the quantitative results of the samples are statistically consistent by using relative standard deviations to evaluate protein quantitative repeatability. As shown in Fig. S3B, the relative standard deviation value of each sample was less than 0.1, indicating that the repeatability of the tested samples was excellent.

### Quantification and annotation of differentially expressed proteins

**Fig. 4:**
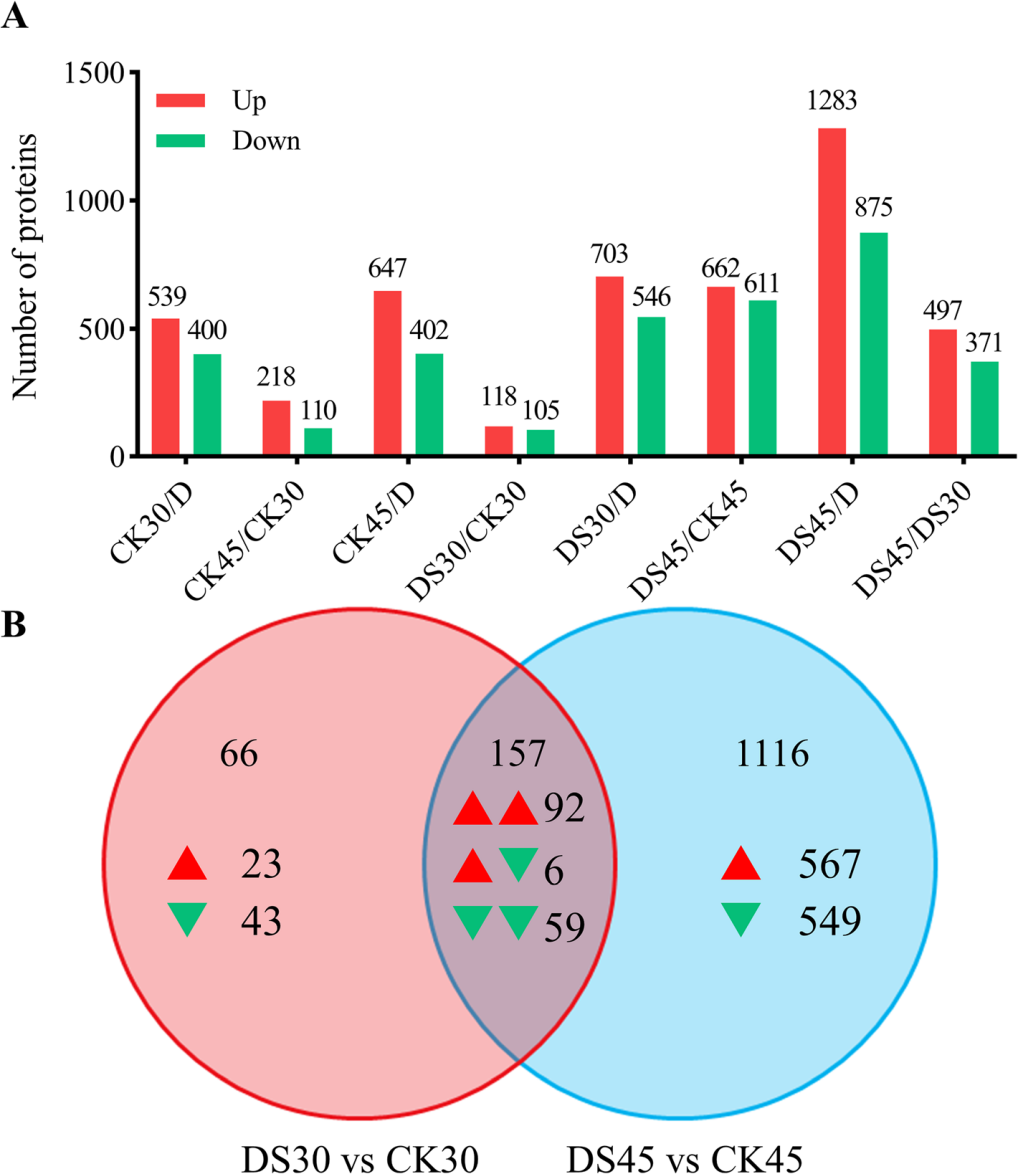
Identification and statistical analysis of differentially expressed proteins (DEPs). **a** Numbers of up- and down-regulated DEPs among eight comparisons. **b** Venn diagram of the number of DEPs in the ‘DS30 vs CK30’ and ‘DS45 vs CK45’ comparisons. Red and green arrowheads represent up- and down-regulated DEPs, respectively

To examine DEPs in response to drought stress, the aforementioned proteome changes at different timepoints in response to drought stress were investigated using eight independent TMT experiments. Among the proteins with significant abundance changes (*P* < 0.05), values showing more than 1.30-fold changes or less than 0.77-fold changes were assessed as DEPs between the comparison groups. The numbers of DEPs that were up- or down-regulated between the eight comparable groups are shown in Fig. 4a. See Table S1 (Additional file 4) for detailed statistical analysis results.

To more thoroughly understand the underlying proteomes of the fine roots of cotton plants under CK and DS treatments, root samples at two critical stress stages (30 and 45 DAD) were selected for TMT proteomic analysis based on the morphological and physiological results mentioned above. We constructed two comparison groups: ‘DS30 vs CK30’ and ‘DS45 vs CK45’; in the former and later comparison groups 223 and 1273 significantly DEPs were revealed, respectively (Fig. 4b). Of those DEPs, 157 were shared between both groups (Additional file 5: Table S2), indicating that these DEPs are only induced by drought stress and may not be related to treatment duration.

### Gene ontology (GO) enrichment analysis of DEPs under drought stress

To uncover the biological mechanisms differentiating the responses to the DS and CK treatments, we annotated the DEPs with GO terms and conducted a GO ‘Biological Process’ enrichment analysis (the enrichment of ‘Molecular function’ and ‘Cellular Component’ are also shown in the figures). See Table S3 (Additional file 6) for detailed statistical analysis results.

Cotton plants at two critical stages (30 and 45 DAD) were subjected to further analysis. For the up-regulated DEPs in the ‘DS30 vs CK30’ comparison, the significantly enriched ‘Biological Process’ GO term was ‘proteolysis’ (GO:0006508) (Additional file 7: Fig. S4A). For the down-regulated DEPs, the most enriched terms in the biological process terms included ‘inorganic anion transport’ (GO:0015698), ‘organic acid biosynthetic process’ (GO:0016053), and ‘anion transport’ (GO:0006820) (Additional file 7: Fig. S4B), indicating that proteins involved in these processes may play pivotal roles in drought sensing.

**Fig. 5:**
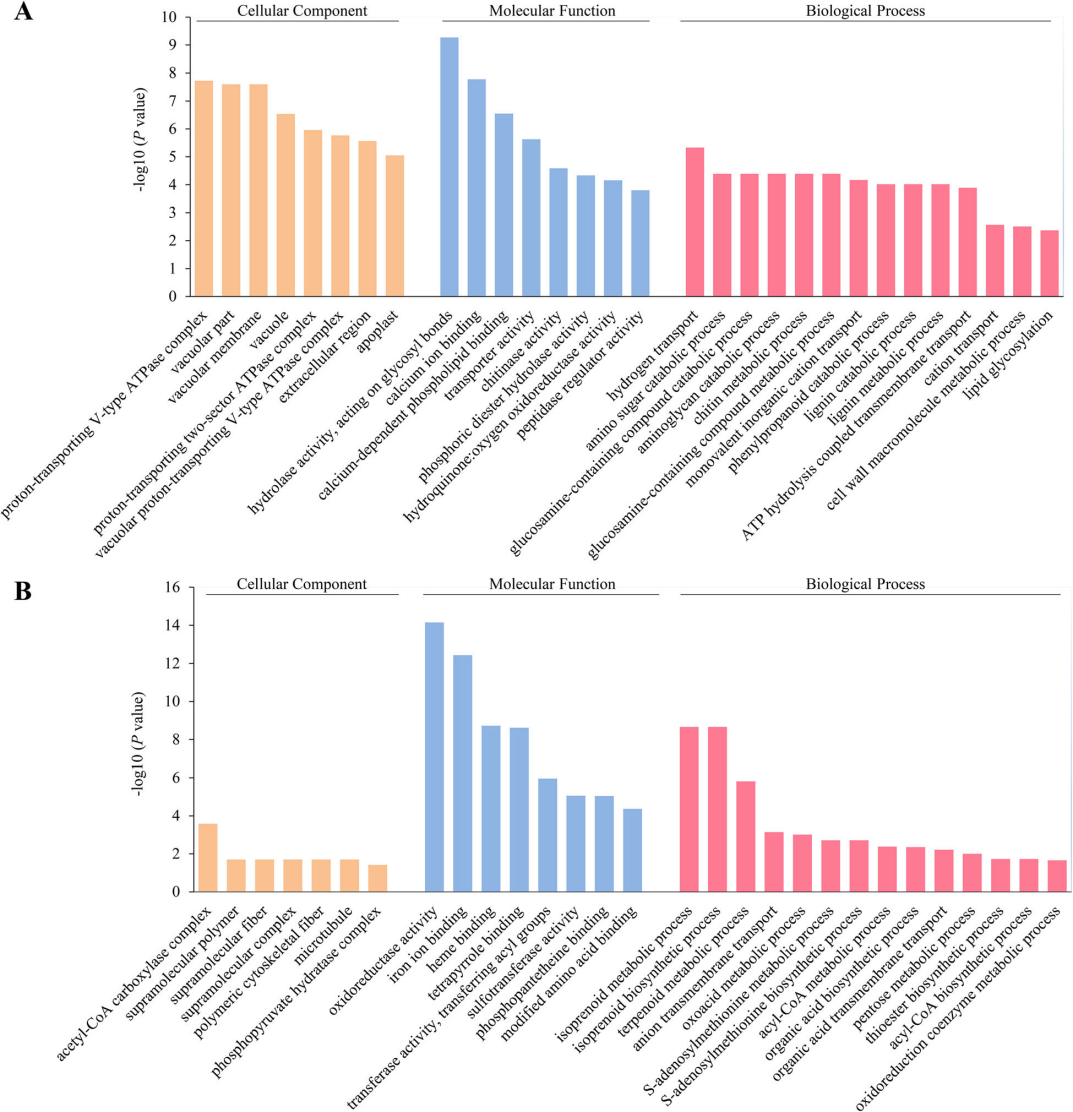
Gene ontology (GO) enrichment analysis of the up-regulated (**a**) and down-regulated (**b**) differentially expressed proteins (DEPs) in the ‘DS45 vs CK45’ comparison

For the up-regulated DEPs in the ‘DS45 vs CK45’ comparison, the significantly enriched biological process terms included ‘hydrogen transport’ (GO:0006818), ‘amino sugar catabolic process’ (GO:0046348), and ‘glucosamine-containing compound catabolic process’ (GO:1901072) (Fig. 5a). For the down-regulated DEPs, the enriched biological process GO terms included ‘isoprenoid metabolic process,’ ‘isoprenoid biosynthetic process,’ and ‘terpenoid metabolic process’ (Fig. 5b).

### KEGG pathway enrichment of DEPs under drought stress

**Fig. 6:**
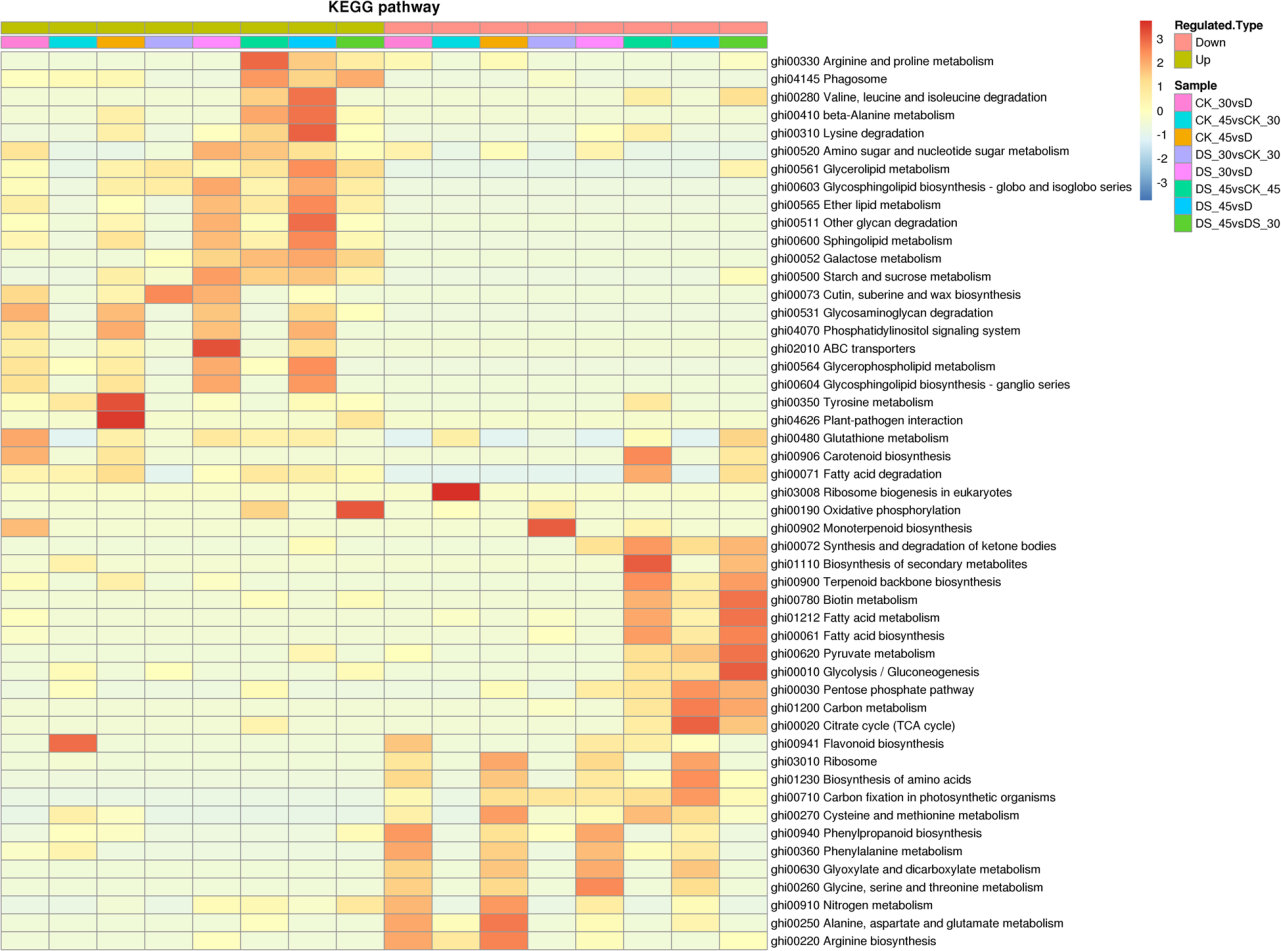
KEGG pathway enrichment-based clustering analysis of all identified proteins

To further understand the function of the DEPs from a pathway-specific perspective, the DEPs among the above eight comparisons were subjected to a Kyoto Encyclopedia of Genes and Genomes (KEGG) pathway enrichment-based clustering analysis in which the main biochemical metabolisms and metabolic pathways of the DEPs involved were described (Fig. 6). See Table S4 (Additional file 8) for detailed statistical analysis results.

The up-regulated DEPs in the ‘DS30 vs CK30’ comparison were mapped onto the ‘Cutin, suberine and wax biosynthesis,’ ‘Glycosphingolipid biosynthesis - globo and isoglobo series,’ ‘Galactose metabolism,’ and ‘Pentose and glucuronate interconversions’ pathways. The down-regulated DEPs were involved in the ‘Monoterpenoid biosynthesis,’ ‘Carbon fixation in photosynthetic organisms,’ and ‘Oxidative phosphorylation’ pathways (Fig. 6; Table S4). The up-regulated DEPs in the ‘DS45 vs CK45’ comparison were most associated with the ‘Galactose metabolism,’ ‘Arginine and proline metabolism,’ and ‘Phagosome’ pathways; the down-regulated DEPs were most enriched in the ‘Biosynthesis of secondary metabolites,’ ‘Fatty acid metabolism,’ and ‘Fatty acid biosynthesis’ pathways (Fig. 6; Table S4).

### Validation of TMT data for selected proteins by parallel reaction monitoring

The protein expression levels obtained by TMT were further confirmed by quantifying the expression levels of 20 proteins using parallel reaction monitoring (PRM) analysis. In detail, 20 DEPs from the ‘DS45 vs CK45’ and ‘DS30 vs CK30’ comparisons were randomly selected.

Among the 20 target proteins assessed, 17 proteins yielded MS/MS spectra and unique peptides. As shown in Table S5 (Additional file 9), the trends of these DEPs determined by PRM were generally consistent with the TMT results. Although there were differences between the two sets of empirical values, the Pearson correlation coefficient between the quantitative results of TMT and PRM was significant (*P* < 0.01), which showed that the results of the two analyses agreed well with each other. The differences in fold changes between the two assays may be attributed to the PRM quantitative analysis having high sensitivity and to the resolution of the analysis of these proteins. Thus, our PRM assay confirmed that the TMT results were credible and meaningful targets for further analysis.

The ion peak area distributions of peptide fragments for all 20 proteins are provided in Additional file 10.

## Discussion

To cope with drought stress, plants have evolved complex strategies by modulating drought-responsive signaling and metabolic processes at the cellular, organ, and whole-plant levels. Fine roots are essential for maintaining water balance under drought stress, and this is achieved, in part. Through the regulation of their proteomes. A detailed assessment of the changes in fine root proteomes in response to drought is essential to understand the mechanisms underlying physiological adaptation to stress. This is the first comprehensive proteomic analysis of the fine roots of cotton plants under drought stress.

### Soil drought affects physiological and growth characteristics

Several studies have shown that drought stress significantly influences the morphology of cotton plants [15, 16]. In this study, plant height, stem diameter, leaf area, and leaf thickness were significantly reduced under soil drought (Fig. S1). This indicated that drought stress severely inhibits the development of the aboveground portions of plants. In addition, we concluded that the SPAD value and LRWC were closely associated with the duration of drought stress (Fig. 2).

The root system coordinates with the aboveground portion of plants by efficiently utilizing limited resources under drought stress, with synergistic action occurring among the portions of plants that regulate growth [17]. Our results showed that the response of root morphology to drought stress in cotton plants was opposite that of the aboveground portions. Under drought stress, fine roots tended to be thinner and longer, thus promoting elongated root systems and the absorption of water from deep in the soil (Fig. S2).

### Effects of soil drought on stress- and defense-related proteins

When plants suffer from drought stress, higher levels of ROS are produced. Furthermore, ROS are used as signal molecules to control programmed cell death, abiotic stress responses, and pathogen defense [18].

Plants have evolved a variety of ROS scavenging strategies to alleviate ROS damage by regulating antioxidant enzyme activities and non-enzyme antioxidant content, which mitigate the adverse effects of drought stress [19]. Among them, SOD provides the first line of defense in antioxidant systems. In this study, the SOD and CAT activities of fine roots began to increase significantly under drought stress compared with CK plants at 30 DAD (Fig. 3). However, the level of DEPs encoding CAT and SOD did not change significantly, indicating that the protein levels were not correlated with their activities. POD is involved in a wide range of physiological processes, including ROS metabolism. Previously, POD levels were shown to be changed under drought stress [18]. In this study, four POD proteins (A0A1U8M415, A0A1U8KFD1, A0A1U8IEZ3, A0A1U8L990) were up-regulated in the ‘DS45 vs CK45’ comparison, consistent with the physiological results observed (Additional File 11: Table S6; Fig. 3b). Ascorbate peroxidase (APX) acts as another type of antioxidant enzyme, and the expression levels of two APX or APX-like proteins (A0A1U8MGX8, A0A1U8L6G4) were significantly up-regulated at 45 DAD (Table S6). Thioredoxin (Trx) is involved in the removal of ROS and is considered a biomarker of oxidative stress [20], and seven Trx or Trx-like proteins (A0A1U8PN19, A0A1U8MVQ7, A0A1U8K8W1, A0A1U8PQ09, A0A1U8IVU7, A0A1U8HYN5, and A0A1U8IT56) were induced at 45 DAD (Table S6). Zhang et al. [21] cloned a Trx superfamily gene *TaNRX* from common wheat (*Triticum aestivum*) and found that it functioned as a drought resistance mechanism, consistent with our findings. Thus, these results confirmed that the soil drought treatment induced up-regulation of active oxygen scavenging enzymes in the fine roots of cotton plants, and long-term drought stress assessed at 45 DAD activated more enzymes than were observed at 30 DAD (Table S6).

The abundance of stress-responsive proteins, especially those related to abiotic stress, such as late embryogenesis abundant (LEA) proteins, germin-like proteins (GLPs), annexin, and heat shock proteins (HSPs) also changed significantly. According to the present proteome data, three LEA proteins (P46518, P09441, A0A1U8L687) were found to be highly up-regulated in the ‘DS45 vs CK45’ comparison (Table S6). Owing to their high hydrophilicity, LEA proteins bind a large number of water molecules, and the accumulation of LEA protein was reported to be closely related to dehydration resistance [22]. Garay et al. [23] found that LEA proteins function as a hydrophilic buffer to reduce the rate of cell water loss under drought stress, thereby ensuring sufficient water remains in tissues in order to maintain normal metabolic activity. GLPs are a type of glycoprotein, and many of them possess the manganese-containing SOD activity that catalyzes ROS into H_2_O_2_ [24]. Previous studies have shown that GLP gene expression was higher under drought stress [25–27]. We found that the abundance of six GLPs (A0A1U8LQB5, A0A1U8PTH1, A0A1U8PBD0, A0A1U8JEA8, A0A1U8P8T2, and A0A1U8L5P7) changed significantly in the ‘DS45 vs CK45’ comparison, while only one (A0A1U8L5P7) was identified in the ‘DS30 vs CK30’ comparison (Table S6). Annexin is a type of protein family that binds to membrane phospholipids in a calcium-dependent manner. Studies have shown that under different stress conditions, the expression of plant annexin will increase and accumulate in large amounts along the cell membrane. It has been speculated that this accumulation may be related to the construction of the ion channel structure, the protection of the cell membrane, and the function of the ROS-induced signal [28–31]. Eight annexin or annexin-like proteins (A0A1U8J8K4, A0A1U8J7H8, A0A1U8IN71, A0A1U8JFY4, A0A1U8I5D0, S5GFP3, A0A1U8JDH4, and A0A1U8J6E7) were up-regulated in the ‘DS45 vs CK45’ comparison, of which six were already identified as up-regulated DEPs at 30 DAD (A0A1U8IN71, A0A1U8J8K4, A0A1U8I5D0, S5GFP3, A0A1U8J6E7, and A0A1U8JDH4) (Table S6). This implies that the response pattern of annexin in fine roots in the early stages of the full drought treatment and after 45 days of soil drought were consistent, suggesting that this upregulation might be a common strategy by which fine roots cope with drought stress. Qiao et al. [29] found that rice annexin OsAnn1 enhanced the tolerance to drought stress and *AtANN1*-deletion *Arabidopsis* mutants showed reduced resistance to drought [28]. In addition, annexin has been reported to be linked to POD activity [32], which was most likely associated with the accumulation of the ROS scavengers APX and POD. As such, annexin likely plays an important role in the process of physiological adaption to drought stress. Furthermore, 13 heat shock protein (HSP) were identified, 5 of which (A0A1U8PG98, A0A1U8MBN0, A0A1U8M2Z7, A0A1U8KRV5, A0A1U8LKC3) were up-regulated (Table S6). HSPs are involved in protein folding and active oxygen clearance, playing a key role in drought stress in cassava (*Manihot esculenta* Crantz) through transcriptional, post-transcriptional, and translational regulation [33]. HSP70 is involved in many cell processes, conferring plant heat tolerance and drought tolerance in both transgenic tobacco and *Arabidopsis* [34], and seven DEPs encoding HSP70 or HSP70-like proteins were identified in this study.

Among the identified DEPs, there were some pathogenesis-related proteins (PRs) identified that showed significant changes under soil drought, such as thaumatin, osmotin, glucan endo-1,3-beta -glucosidase, and chitinases [35, 36]. In the ‘DS45 vs CK45’ comparison, ‘pathogenesis-related protein PR-4A-like’ was significantly up-regulated (Table S6). PR proteins can be divided into 18 families (PR-1 to PR-18). Among them, PR-5 protein, also known as thaumatin-like protein (TLP), is accumulated rapidly when plants are subjected to different stresses, with accumulation being significantly correlated with the intensity of plant stress [37]. Osmotin also belongs to the PR-5 family, and it has a structure similar to that of TLP. According to the present study, ‘osmotin-like protein’ (A0A1U8PK21) and ‘osmotin-like protein I’ (Q2HPG3) were 1.48- and 2.20-fold up-regulated at 45 DAD. Glucan endo-1,3-beta-glucindase belongs to the PR-2 family, which is involved in cell division, flower formation, seed maturation, and plant responses to abiotic stress [38]. Our results revealed that three ‘glucan endo-1,3-beta-glucosidase-like’ proteins (A0A1U8N331, A0A1U8HW63, A0A1U8I6K1) were up-regulated (Table S6) at 45 DAD. Chitinase is another kind of well-characterized pathogen-related protein [38]. The chitinase family can be divided into sub-families that include endochitinases, exochitinases, β-*N*-acetylglucosidases, and chitosidases. These enzymes work together to gradually degrade chitin into monosaccharides and enhance plant defense against abiotic stress [39]. In this study, three DEPs (A0A1U8PCX3, A0A1U8IHA4, Q39799) encoding endochitinases were up-regulated (Table S6).

Taken together, the elevated levels of these antioxidants and pathogenesis-related proteins are likely strategies for cotton plants to cope with the deleterious effects of ROS, and increased stress durations are likely to activate the expression of more related proteins.

### Effects of soil drought on ion transport-related proteins

Maintenance and re-establishment of cellular ion homeostasis during stress conditions is extremely important for plant survival and growth, especially for plants under osmotic stressors such as drought [40]. In the current study, it was found that a certain number of DEPs were enriched among the biological process terms related to ion transport, including ‘hydrogen transport’ (GO:0006818), ‘monovalent inorganic cation transport’ (GO:0015672), ‘cation transport’ (GO:0006812), and ‘anion transmembrane transport’ (GO:0098656) (Table S3). This has aroused great interest in the analysis of the DEPs involved. (Additional file 12: Table S7).

V-type ATPases transport hydrogen ions to the vesicles or extracellularly, thus maintaining a stable acid–base environment in cells [41]. Overexpression of *AVP1* (a gene encoding a protein that can generate a H^+^ gradient across the vacuolar membrane similar in magnitude to that of the multisubunit vacuolar H^+^-ATPase) in transgenic *Arabidopsis* substantially increased resistance to drought relative to wild-type plants, and it was also found that the resistant phenotypes had increased vacuolar proton gradients, resulting in increased solute accumulation and water retention [42]. V-type ATPase is also induced in the roots of *Arabidopsis* [43], wheat [44], and cucumber [45] under abiotic stress conditions. Here, the increased abundance of twelve V-type proton ATPases in this study indicates that the increased activities of these enzymes are considered to be a cost-effective strategy for coping with long-term stress (i.e., at 45 DAD) (Table S7). Overexpression of the V-ATPase G subunit in walnut (*Juglans regia*) effectively improved drought resistance in transgenic plants [46]. In contrast with the 45 DAD results, we found two ‘V-type proton ATPase subunit G’ (A0A1U8KTX7 and A0A1U8NHE1) were down-regulated in the ‘DS30 vs CK30’ comparison, which may indicate that there are two completely opposite regulatory strategies for V-type ATPases between 30 and 45 DAD.

ABC transporters transport stress-related secondary metabolites such as alkaloids, terpenoids, polyphenols, and quinines [47]. In the current study, five ABC transporters (A0A1U8PJL1, A0A1U8N9K0, A0A1U8L5Z3, A0A1U8LYD7, and A0A1U8KBE6) were found to be induced by soil drought in the ‘DS45 vs CK45’ comparison, while in the ‘DS30 vs CK30’ comparison, only one ABC transporter (A0A1U8L5Z3) was identified as a DEP. This confirmed the findings of a previous study in which ABC transporters were shown to improve the resistance of crops to abiotic stresses such as drought [48], and this may indicate that the up-regulation of ABC transporters in the fine roots of cotton plants is an effective regulation strategy under soil drought. Kim et al. [48] also found that overexpression of *AtABCG36*, an ABC transporter gene in *Arabidopsis*, can greatly increase the ability of ABC transporter to transport sodium ions and significantly improved the drought resistance of Arabidopsis plants, supporting our hypothesis.

Plants have complex systems used to absorb and transport nitrogenous compounds (e.g., nitrate, ammonium, oligopeptides, and amino acids). The nitrate transport gene family is divided into low- and high-affinity transport families. When the concentration of nitrate in the outside world is less than 0.5 mM, the high-affinity transport family is mainly responsible for its function [49]. In this study, ‘high-affinity nitrate transporter 2.1 (A0A1U8PWK7)’ and ‘high-affinity nitrate transporter 3.2 (A0A1U8NYW8)’ were down-regulated at 30 DAD. In order to better absorb, utilize, and distribute nitrate, plants have evolved different transport vectors or channel proteins to cope with environmental changes. The genes encoding these proteins are mainly divided into four families: NRT1, NRT2, CLC, and SLAV1/SLAH. Of these families, only NRT1 and NRT2 participate in the absorption of nitrate by roots, and they were later collectively renamed NRT1/PRT FAMILY (NPF) proteins according to their evolutionary history [50]. Taochy et al. [51] found that AtNPF2.3 was responsible for transporting nitrate from roots to shoots when plants were subjected to salt stress. In the current study, five NRT1/ PTR FAMILY proteins (A0A1U8PIU8, A0A1U8LJU9, A0A1U8MGW2, A0A1U8MWX1, and A0A1U8M2N9) were down-regulated at 45 DAD. This shows that long-term drought stress may hinder the absorption of nitrate by the fine root system, which in turn leads to a reduction in the nitrates allocated to the aboveground portions of plants. Additionally, some recent studies have revealed that NPF proteins also transport plant hormones, including auxin, abscisic acid, jasmonates, and gibberellin [52, 53].

It is generally believed that the input of drought signals depends on the mechanical load of the membrane. Changes in mechanically sensitive ion channel activity can sense changes in cell membrane tension due to loss of turgidity [54]. In plant cells, such mechanosensitive channels drive an influx of calcium [55]. In this study, two mechanosensitive ion channel proteins (A0A1U8NHU2, A0A1U8LSY1) and one calcium-transporting ATPase were also greatly induced at 45 DAD. It was also observed that five syntaxin-like proteins were exclusively induced by soil drought. Some studies have demonstrated that syntaxin proteins can interact with and coordinate the trafficking of plasma membrane aquaporin to modulate the water permeability of cell membranes [56, 57].

### Short-term soil drought affects ‘Cutin, suberin and wax biosynthesis’ in cotton fine roots

The most enriched KEGG pathway in the ‘DS30 vs CK30’ comparison was ‘Cutin, suberin and wax biosynthesis,’ and it was not enriched at 45 DAD (Fig. 6), indicating that this pathway likely plays an important role when cotton fine roots respond to early drought and attracting our focus.

During the evolution of land plants, epidermal tissues evolved to prevent the loss of water and nutrients [58]. These epidermal tissues are composed of cutin, wax, and inner suberin layers [59]. Suberin is ubiquitous in specific internal root-tissues, where it controls water and ion uptake and also play roles in protecting plants from abiotic stresses and establishing plant morphology [60]. In the current study, the three up-regulated DEPs enriched in the ‘Cutin, suberin and wax biosynthesis’ pathway were ‘peroxygenase-like’ (A0A1U8HRH9), ‘probable peroxygenase 5 isoform X2’ (A0A1U8K2T6), and ‘omega-hydroxypalmitate *O*-feruloyl transferase-like’ (A0A1U8HNM9). Suberin is composed of suberin polyphenolic and polyaliphatic domains. Omega-hydroxypalmitate *O*-feruloyl transferase (HHT) is the main enzyme that regulates the phenylpropane-ferulic acid pathway, which directly or indirectly affects the expression of ferulic acid, thus affecting the structural composition of the suberin polyphenolic and polyaliphatic domains. Lotfy et al. [61] have shown that HHT can promote suberin formation in potato; *Arabidopsis esb1* (*enhanced suberin 1*) mutants have increased suberin content and increased their water use efficiency during their vegetative growth stage, resulting in increased resistance to wilt relative to wild-type plants under drought stress [62]. Our TMT results suggest that the response of fine roots of cotton plants to the 30-days soil drought treatment is likely to increase the content of suberin by up-regulating HHT expression, thereby reducing water loss. Peroxygenase is a key enzyme involved in the formation of cutin [63]. Maize treatment with peroxygenase inhibitor caused cuticle changes, resulting in increased permeability to pesticides [63]. Therefore, it is likely that proteins belonging to the ‘Cutin, suberin and wax biosynthesis’ pathway were activated in the fine roots of cotton plants during the early stages of soil drought, thus promoting increased suberization of epidermal tissues, so as to protect internal vascular tissues from drought stress. This, in turn, would maintain the vascular connection between the root system and shoots, helping plants resist short-term drought. However, we did not find that this pathway was enriched in the ‘DS45 vs CK45’ comparison, suggesting that drought stress caused functional damage to the fine root epidermis at this stage.

### Long-term soil drought activated more phytohormone-related DEPs than short-term drought

According to the physiological and morphological results of our experiment, 45 days of soil drought caused serious damage to cotton plants. To explore the mechanisms of drought stress in fine roots at 45 DAD, we performed differential proteomic analysis between fine roots under the CK and DS treatments. Compared with the ‘DS30 vs CK30’ comparison, there were more identified DEPs and more enriched metabolic pathways at 45 DAD. We classified these pathways into five categories based on their first-level KEGG classification, including ‘Lipid metabolism,’ ‘Secondary metabolism,’ ‘Energy metabolism,’ ‘Carbohydrate metabolism,’ and ‘Amino acid metabolism,’ each of which has been widely studied in previous articles. Some DEPs that play an important role are listed in Table S8 (Additional file 13).

A series of adaptive responses produced by plants under drought stress are controlled by many phytohormones, and they are thus the basic mediators for tolerating or avoiding the negative effects of water deficit. In the current study, some important DEPs were identified to be involved in the regulation of phytohormones at 45 DAD, and only a small part of these DEPs appeared in the ‘DS30 vs CK30’ comparison (Additional file 14: Table S9).

One of the proteins markedly up-regulated by drought at 45 DAD was identified as indole-3-acetic acid-amido synthetase GH3.17-like protein (Table S9). The proteins of the *GH3* family have hormone amide synthetase activity, catalyzing the binding of free auxin (IAA) to amino acids [64]. *OsGH3.13* encodes indole-3-acetic acid-amino synthetase in rice, which improves plant drought resistance [65]. *S*-adenosylmethionine synthetase (SAMS) functions as one of the key enzymes in the ethylene synthesis pathway [66]. Four SAMS (A0A1U8P2T2, A0A1U8L5H6, A0A1U8JUM7, A0A1U8NVJ7) and five 1-aminocyclopropane-1-carboxylate oxidases (A0A1U8NWE4, A0A1U8MU28, A0A1U8JC48, A0A1U8JY55, A0A1U8PRG1) were identified as down-regulated DEPs at 45 DAD. As the last enzyme in the ethylene pathway, 1-aminocyclopropane-1-carboxylate oxidase (ACO), is generally considered the rate-limiting enzyme in ethylene biosynthesis [67]. A large amount of ACO is induced under drought conditions, which decomposes 1-aminocyclopropane-1-carboxylate into ethylene, eventually leading to an increase in the expression of ACO genes and ethylene production [68, 69]. However, the results we obtained were contrary to earlier research, suggesting that the fine roots of cotton plants activated a response mechanism when challenged by drought, which led to a decline in ACO levels.

It has been shown that the interaction between IAA and ABA promotes the development of lateral roots in plants, and the morphology of roots is a necessary element of plant responses to drought stress [70]. Some proteins involved in abscisic acid (ABA) metabolic were also identified as DEPs in this study. Abscisic acid 8′-hydroxylase (ABAH) acts as the key enzyme in the ABA oxidative inactivation pathway [71]. Takeuchi et al. [72] reported that ABAH inhibitors can significantly improve drought tolerance in *Arabidopsis*. In the current study, it was determined that two ABAH proteins (A0A1U8NTQ2, A0A1U8N913) were down-regulated at 45 DAD (Table S9), indicating that the fine roots of cotton plants activate a corresponding drought resistance mechanism by down-regulating ABAH protein expression. ABSCISIC ACID-INSENSITIVE 5 (ABI5) is a key factor involved in ABA response, and its protein stability and protein phosphorylation are all regulated by ABA, with different degrees of increases exhibited under abiotic stresses [73–75]. In this study, two ABI5 proteins (A0A1U8PG85, A0A1U8P7L8) were found to be significantly up-regulated at 45 DAD, confirming previous proteomic studies [73–75].

The above results indicated that the fine roots of cotton were activated across a series of signal transmission pathways under long-term drought stress, some of which are involved in the regulation of phytohormones, and may therefore eventually lead to changes in phytohormone levels.

**Fig. 7:**
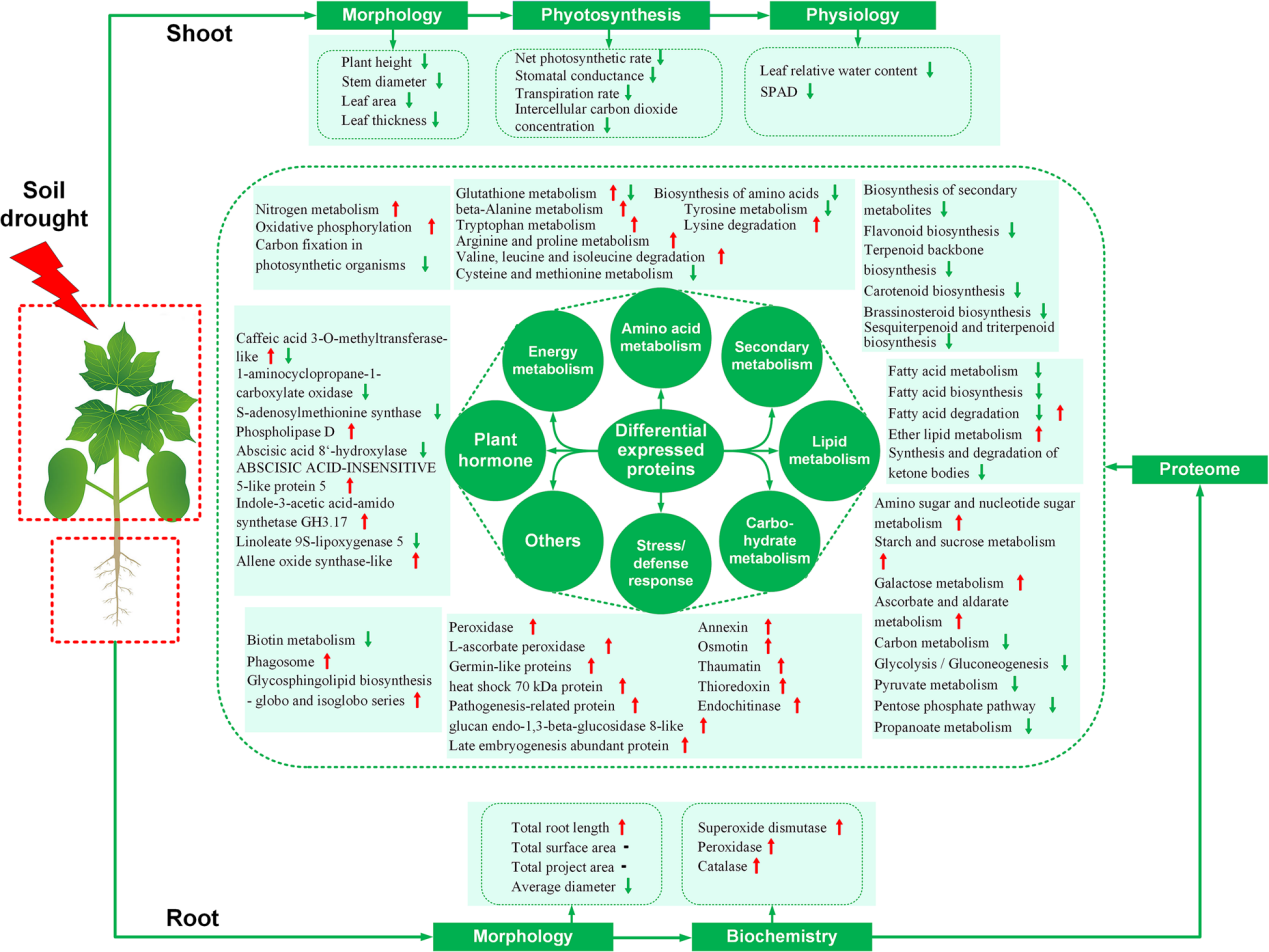
Model showing the responses of the fine roots of cotton plants

Based on our results and previous studies, strategies to minimize the harm of drought stress on cotton plants or improve the resistance of cotton plants to drought stress can be determined. In Fig. 7, we summarize the response of cotton fine roots to 45 days of soil drought based on the above morphological, physiological, and proteomic results. First, selecting cotton varieties with longer root systems and growing cotton in soil types that facilitate root penetration are effective strategies for enhancing the adaptability of cotton plants to drought conditions. Second, exogenous application of plant hormones or growth regulators with similar effects can be an effective method of improving drought resistance of cotton plants. Finally, based on results from different stress stages, appropriate proteins can be identified for the purpose of altering the genetics of crops through traditional artificial selection or genetic transformation. In short, our results enhance the current understanding of the protein expression mechanism in the fine roots of cotton plants under drought stress and provide new targets for genetic improvement and enhanced agronomic management practices.

## Conclusions

Our investigation conducted in cotton indicates that drought treatment restricted the development of aboveground tissues, but the development of root systems exhibited a contrasting pattern; the root system became more active under drought stress by adopting a longer and thinner architecture. TMT-based proteomic techniques were applied to compare the abundance of proteins in fine roots under normal irrigation and drought-stressed conditions at 30 and 45 DAD. The proteins were classified into a broad range of pathways, with a particular enrichment of those participating in carbohydrate and energy metabolism, fatty acid metabolism, amino acid metabolism, and biosynthesis of secondary metabolites. We also identified DEPs in fine roots under drought stress, many of which are related to stress/defense response and plant hormone metabolisms. Overall, our proteome profiling identified key pathways and proteins that are involved in plant responses to drought stress, which may serve as the basis for improving drought tolerance in cotton and other plants.

## Methods

### Plant materials and drought treatments

The experiments were conducted during the 2018 and 2019 growing seasons at Hebei Agricultural University (38°85′N, 115°30′E, Hebei Province, China), which is located in the Yellow River basin. ‘Guoxin 9,’ a commercial cotton (*Gossypium hirsutum* L.) cultivar with high-yielding potential, was used in the experiment. The cultivar was developed by Guoxin Rural Technical Service Association and officially registered and released by the Chinese Crop Cultivar Registration Committees (GSM2009004).

Seeds were surface-sterilized by soaking in a 5% (*v*/*v*) sodium hypochlorite solution for 15 min followed by three washes with sterile distilled water and germination in an incubator at 25 °C in the dark for 24 h. The germinated seeds were sowed into pots containing a mixture of topsoil (sampled from the top 20-cm layer of soil of the cotton experiment field of Hebei Agricultural University; pH 7.20; organic matter content, 10.0 mg·kg^− 1^; total N, 1.23 g·kg^− 1^; alkali-hydrolyzable N, 37.6 mg·kg^− 1^; available phosphorus, 10.9 mg·kg^− 1^; available potassium, 109 mg·kg^−− 1^), nutrient soil (Pindstrup Plus, Ryomgaard, Denmark; pH 6.0; screened to particle sizes of 0–6 mm), and vermiculite (3:1:1, *v*/*v*/*v*; one plant per 1.8-L pot with a 15-cm diameter) in an environmentally controlled greenhouse. The seedlings were cultured under the following growth conditions: 30/25 °C (day/night), 40–45% relative humidity, and a photoperiod of 16 h/8 h (day/night) provided by 600 μmol m^− 2^ s^− 1^ light intensity during the daytime phase. At the three-true-leaf stage, the seedlings were subjected to various water supply treatments, including normal irrigation (CK) treatment, with a sustained soil relative water content of 70–75%, and drought stress (DS) treatment, with a soil relative water content of 40–45%. The pots were randomized in 50 replicates between the two treatments. All of the pots were maintained within the required soil moisture range by weighing pots every other day.

Fine root samples were collected 0, 15, 30, 45, and 60 DAD. After collection, a portion of the fine roots was immediately frozen in liquid nitrogen and stored at − 80 °C for proteomic analyses. Another portion of each sample was used for measurement of biochemical indicators.

### Measurements of aboveground morphological traits

The morphological indicators of the aboveground portions of cotton plants were evaluated at five time points (0, 15, 30, 45, and 60 DAD). Plant heights were measured using a ruler; the leaf areas were calculated according to the length–width coefficient method; the stem diameter and the thickness of main stem leaves were assessed using Vernier calipers. The average values of the traits mentioned above were obtained from five replicates.

### Assay of root morphological traits

The roots of cotton plants grown in pots were washed clean by slowly rinsing away soil with running water along the edge of the pot, which removed soil particles attached to the roots. This was performed carefully to avoid damaging the roots. After being rinsed, the roots were separated from the above-ground tissues of plants and then immersed in a plexiglass tank containing 3–5 mm of clear water; images were then captured after separation of root tissues using medical tweezers. The roots were also scanned after images were taken using a double-sided light source scanner (EPSON Perfection V700 Photo, Suwa, Japan) at 600 dpi. WinRHIZO Reg 2009c software (Regent Instruments Inc., Quebec, Canada) was used to automate measurements of the overall root morphology indicators, including total root length, total surface area, average root diameter, and total projected area. Data were derived from five replicates per treatment.

### Measurement of leaf relative water content

Leaf relative water content (LRWC) in functional leaves was measured 0, 15, 30, 45, and 60 DAD, according to the method described by Barrs and Weatherley [76]. The fresh weight (FW) measures were immediately made after sampling, and then samples were immersed in distilled water for 4 h (TW) at room temperature (25 °C). The leaf samples were then blotted dry and weighed after being oven-dried at 85 °C for 48 h (DW). The LRWC was calculated based on the following formula: LRWC (%) = [(FW − DW) / (TW − DW)] × 100%. All measurements were replicated five times per plot, with each measurement using different plants.

### Chlorophyll content

Leaf chlorophyll contents of the third leaf from the top of each plant were determined at 0, 15, 30, 45, and 60 DAD based on the soil and plant analyzer development (SPAD) values assessed using a SPAD analyzer (SPAD-502, Konica-Minolta, Tokyo, Japan). The recorded SPAD value was derived from the averages of leaves at the base, middle, and top of each leaf, with major veins avoided for each measurement. The average SPAD value in each treatment was calculated across five replicates.

### Measurement of enzymatic antioxidant activity levels

Fine roots (0.5 g) of cotton plants grown under the CK and DS treatments at 0, 15, 30, 45, and 60 DAD were used in this experiment. Superoxide dismutase (SOD), catalase (CAT), and peroxidase (POD) activity levels were determined using assay kits (Nanjing Jiancheng Bioengineering Institute, Nanjing, China) according to the manufacturer’s instructions.

### Protein extraction, trypsin digestion, and TMT labeling

Proteins were extracted from fine root samples from cotton plants using the phenol extraction method. Samples were removed from storage at − 80 °C, weighed, and added to a mortar pre-cooled with liquid nitrogen, and liquid nitrogen was added to each sample prior to it being fully ground into powder. Each group of samples was combined with four times the volume of powdered phenol extraction buffer (containing 10 mM dithiothreitol, 1% protease inhibitor, and 2 mM EDTA) and sonicated. An equal volume of Tris-balanced phenol was added prior to centrifugation at 5500×*g* for 10 min at 4 °C. To the supernatant was added five volumes of 0.1 M ammonium acetate/methanol, and the solution was allowed to form a precipitate overnight. The protein precipitate was washed with methanol and acetone, respectively. Finally, the pellet was reconstituted with 8 M urea, and the protein concentration was measured using a BCA kit (Bio-Rad protein assay kit, Bio-Rad, Hercules, CA, USA) according to the manufacturer’s instructions.

The protein solutions were reduced with 5 mM dithiothreitol for 30 min at 56 °C and alkylated with 11 mM iodoacetamide for 15 min at room temperature in darkness. The urea concentration of the samples was diluted to below 2 M, and pancreatin was added at a ratio of trypsin to protein of 1:50 (*m*/*m*). Samples were then allowed to incubate overnight at 37 °C for trypsin digestion. Trypsin was added to the solution at a ratio of 1:100 (*m*/*m*), and digestion was performed again for 4 h. After that, the fine root peptides obtained from digestion were desalted using a Strata X C18 column (Phenomenex, Torrance, CA, USA) and then vacuum freeze-dried. The peptides were thoroughly dissolved with 0.5 M TEAB. The sample peptides were labeled according to the TMT kit instructions. After the labeling reagents were fully thawed, they were dissolved in acetonitrile, mixed with the peptides, and incubated for 2 h at room temperature. The resulting labeled peptides were mixed, desalted and freeze-dried under vacuum.

### LC-MS/MS analysis and database search

Before mass spectrometry (MS) analysis, the tryptic peptides were fractionated into fractions by high-pH reverse-phase HPLC with an Agilent 300Extend C18 column (5 μm particles, 4.6 mm ID, 250 mm length; Agilent, Santa Clara, CA, USA).

The step gradient of the peptide was set to 8%–32% acetonitrile (pH 9.0), and separate 60 fractions in 60 min, then combine the peptides into nine fractions and freeze-dry in vacuum.

Before MS analysis, the mobile phase needs to be prepared. Solvent A was an aqueous solution containing 0.1% formic acid and 2% acetonitrile. The required formic acid content in solvent B solution was the same as solvent A, but the concentration of acetonitrile needs to be changed to 90%.

The peptides were dissolved in solvent A and separated by EASY-nLC 1200 UPLC system (Thermo Fisher Scientific, Waltham, MA, USA). The liquid phase gradient was set to: 9% − 25% B, 30 min; 25% − 35% B, 22 min; 35% − 80% B, 4 min; 80% B, 4 min, all at a constant flow rate of 350 nL/min. The peptides were then subjected to a NSI source, and tandem mass spectrometry (MS/MS) was performed on an Orbitrap Fusion LUMOS platform coupled online to the UPLC. The MS parameters to be set were electrospray voltage (2.0 kV), primary MS scan range (350–1550 m/z), resolution of primary MS (60,000), fixed starting point for secondary MS scanning (100 m/z), resolution of secondary MS (15,000), data acquisition mode (data-dependent), automatic gain control (5E4), signal threshold (50,000 ions/s), maximum injection time (70 ms) and dynamic tandem mass spectrometry exclusion time (30 s).

The secondary MS data was retrieved using MaxQuant (http://www.maxquant.org/). Tandem mass spectra were searched against the *Gossypium hirsutum*_UniProt database (76,175 sequences) combined with a reverse decoy database to calculate the false positive rate (FDR) caused by random matches. Up to two trypsin/P cleavages were permitted to be missing. The mass tolerance for precursor ions was set at 20 ppm in the first search and 5 ppm in the main search. For fragment ions, it was set at 0.02 Da. The quantitative method was set to TMT-6plex, and the FDR for protein identification and PSM identification was adjusted to < 1%.

### Functional classification of proteins

The functional annotation of the DEPs was annotated to Gene Ontology (GO) and Kyoto Encyclopedia of Genes and Genomes (KEGG) databases. The DEPs ID were converted to UniProt ID, and then the UniProt ID were used to match the GO ID, and the corresponding information would be retrieved from the UniProt-GOA database (http://www.ebi.ac.uk/GOA/) according to the GO ID. If there were no DEPs information in the UniProt-GOA database, InterProScan (an algorithm software based on protein sequence) would be used to predict the GO function of the DEPs. These DEPs were then classified according to three categories: biological process, molecular function, and cellular component. The KEGG database (http://www.genome.jp/kegg/) was used to classify and group the identified DEPs. Fisher’s exact two-terminal test was used to test the DEPs against the background of the identified proteins. Significance was calculated at a *P* < 0.05 threshold for the pathway enrichment test.

### Parallel reaction monitoring (PRM) analysis

To determine the reliability of TMT results, a PRM assay was performed using the original protein samples. Twenty significant DEPs were randomly selected for PRM analysis. Protein extraction and tryptic digestion were performed in the same way as in the TMT experiment. Detailed procedures are provided in Methods S1 (Additional file 15).

### Statistical analyses

Statistical analyses for morphological, physiological, and biochemical results were performed across five biological replicates and for proteomic analyses across three biological replicates. Analysis of variance (ANOVA) was performed using IBM SPSS Statistics 26.0 (IBM Corp., Armonk, NY, USA). Data are presented as means ± standard deviation (SD) values. The statistical significance of Student’s *t*-tests was determined at a *P* < 0.05 threshold.

## Supplementary information

**Additional file 1: Figure S1.** Aboveground morphological responses to drought. Changes of palnt height (A), stem diameter (B), total leaf area (C), and leaf thickness (D) of cotton during stress. Each data point represents the mean of five independent biological replicates (mean ± SD). *Represents a statistically significant difference when compared with the control (*P < 0.05; **P < 0.01).

**Additional file 2: Figure S2.** Root morphological responses to drought. Changes of total root length (A), total average diameter (B), total surface area (C), and total project area (D) of cotton during drought stress. Each data point represents the mean of five independent biological replicates (mean ± SD). *Represents a statistically significant difference when compared with the control (*P < 0.05; **P < 0.01).

**Additional file 3: Figure S3.** Length distribution of peptides identified by mass spectrometry (A); distribution of protein quantitative relative standard deviation (RSD) among repeated samples (B).

**Additional file 4: Table S1.** Proteins identified and quantified via a TMT proteomics-based approach in ‘DS45/DS30’ comparison group.

**Additional file 5: Table S2.** Common DEPs between ‘DS30 vs CK30’ and ‘DS30 vs CK30’ comparison groups.

**Additional file 6: Table S3.** GO enrichment of DEPs in ‘DS45 vs DS30’ comparison group.

**Additional file 7: Figure S4.** Gene ontology (GO) enrichment analysis of the differentially expressed proteins (DEPs) in “DS30 vs CK30” comparsion group. (A) Up-regulated DEPs and (B) down-regualted DEPs.

**Additional file 8: Table S4.** KEGG pathway enrichment of DEPs in ‘DS45 vs DS30’ comparison group.

**Additional file 9: Table S5.** Comparison between the isobaric labels tandem mass tags (TMT) and parallel reaction monitoring (PRM) results.

**Additional file 10:.** Distributions of peptide fragment ion peak area.

**Additional file 11: Table S6.** List of some important DEPs related to stress and defense response.

**Additional file 12: Table S7.** List of some important DEPs related to ion transport.

**Additional file 13: Table S8.** List of some important DEPs involved in the enriched pathways in ‘DS45 vs CK45’ comparison group.

**Additional file 14: Table S9.** List of some important DEPs related to phytohormones.

**Additional file 15: Method S1.** Parallel reaction monitoring (PRM) analysis procedure

## Data Availability

The mass spectrometry proteomics data have been deposited to the ProteomeXchange Consortium via the PRIDE partner repository with the dataset identifier PXD018692 (http://www.ebi.ac.uk/pride/archive/projects/PXD017736).

## Abbreviations

ABA: Abscisic acid
ABAH: Abscisic acid 8′-hydroxylase
ABI5: ABSCISIC ACID-INSENSITIVE 5
ACO: 1-aminocyclopropane-1-carboxylate oxidase
AOS: Allene oxide synthase
APX: Ascorbate peroxidase
CAT: Catalase
DAD: Days after drought stress
DEPs: Differentially expressed proteins
DS: Drought stress
GLPs: Germin-like proteins
GO: Gene Ontology
HSP: Heat shock protein
JA: Jasmonic acid
KEGG: Kyoto Encyclopedia of Genes and Genomes
LEA: Late embryogenesis abundant
LRWC: Leaf relative water content
POD: Peroxidase
PRs: Pathogenesis-related proteins
PRM: Parallel reaction monitoring
ROS: Reactive oxygen species
SAMS: *S*-adenosylmethionine synthetase
SOD: Superoxide dismutase
SPAD: Soil and plant analyzer development
TLPs: Thaumatin-like proteins
TMT: Tandem mass tags.
