# Supplementary material for: Tandem mass tag-based (TMT) quantitative proteomics analysis reveals the response of fine roots to drought stress in cotton (Gossypium hirsutum L.)
Source: BMC Plant Biol. 2020 Jul 11;20:328. doi: 10.1186/s12870-020-02531-z (PMC7353779; doi:10.1186/s12870-020-02531-z)
Supplement: Supplementary file 1 — Additional file 1: Figure S1. Aboveground morphological responses to drought. Changes of palnt height (A), stem diameter (B), total leaf area (C), and leaf thickness (D) of cotton during stress. Each data point represents the mean of five independent biological replicates (mean ± SD). *Represents a statistically significant difference when compared with the control (*P < 0.05; **P < 0.01). [file 12870_2020_2531_MOESM1_ESM.pdf]

Fig. S1

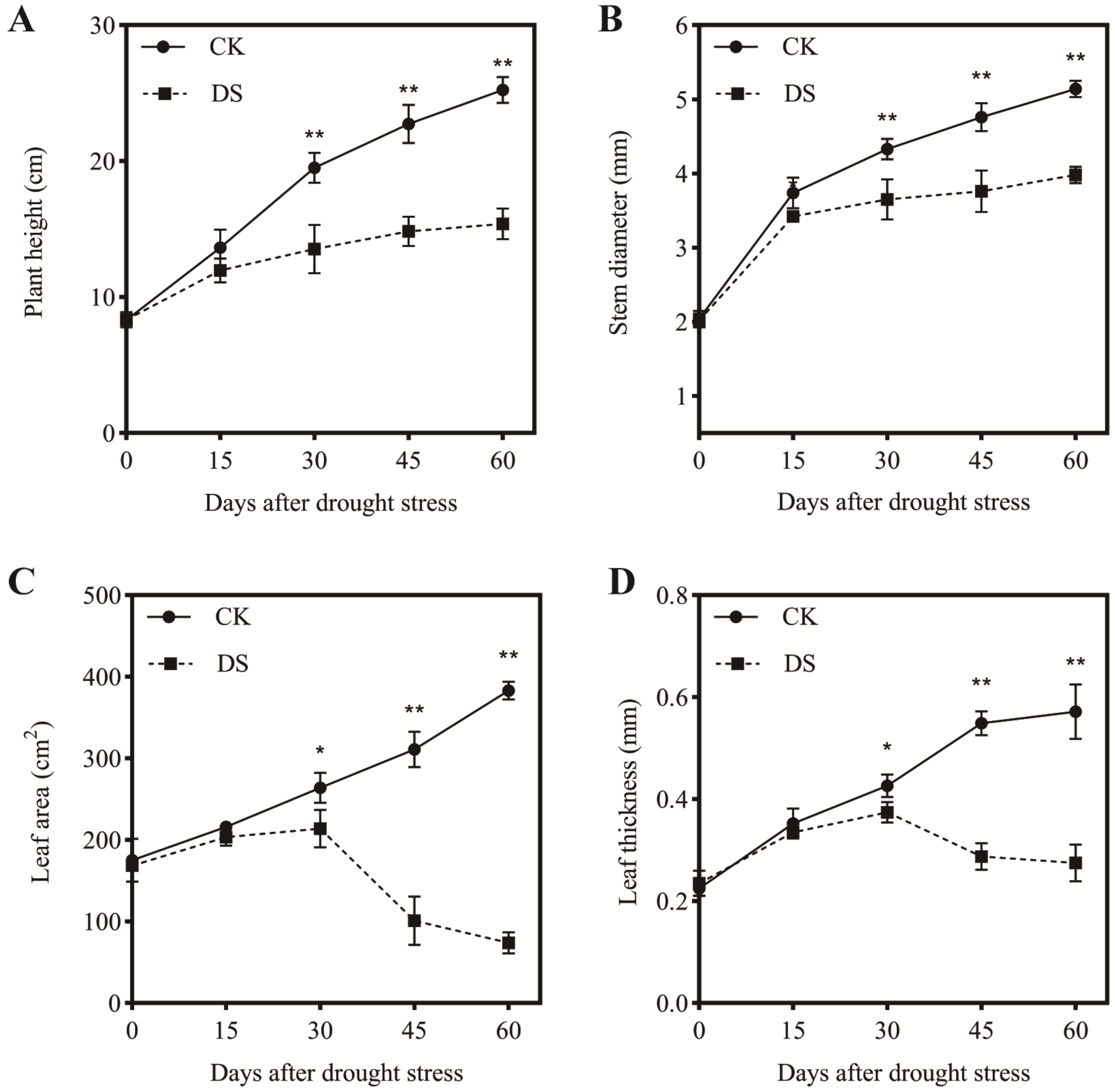

Additional file 1: Fig. S1. **Aboveground morphological responses to drought.** Changes of plant height (A), stem diameter (B), total leaf area (C), and leaf thickness (D) of cotton during drought stress. Each data point represents the mean of five independent biological replicates (mean± SD). *Represents a statistically significant difference when compared with the control (* P<0.05; ** P<0.01).
