# Supplementary material for: Tandem mass tag-based (TMT) quantitative proteomics analysis reveals the response of fine roots to drought stress in cotton (Gossypium hirsutum L.)
Source: BMC Plant Biol. 2020 Jul 11;20:328. doi: 10.1186/s12870-020-02531-z (PMC7353779; doi:10.1186/s12870-020-02531-z)
Supplement: Supplementary file 2 — Additional file 2: Figure S2. Root morphological responses to drought. Changes of total root length (A), total average diameter (B), total surface area (C), and total project area (D) of cotton during drought stress. Each data point represents the mean of five independent biological replicates (mean ± SD). *Represents a statistically significant difference when compared with the control (*P < 0.05; **P < 0.01). [file 12870_2020_2531_MOESM2_ESM.pdf]

Fig. S2

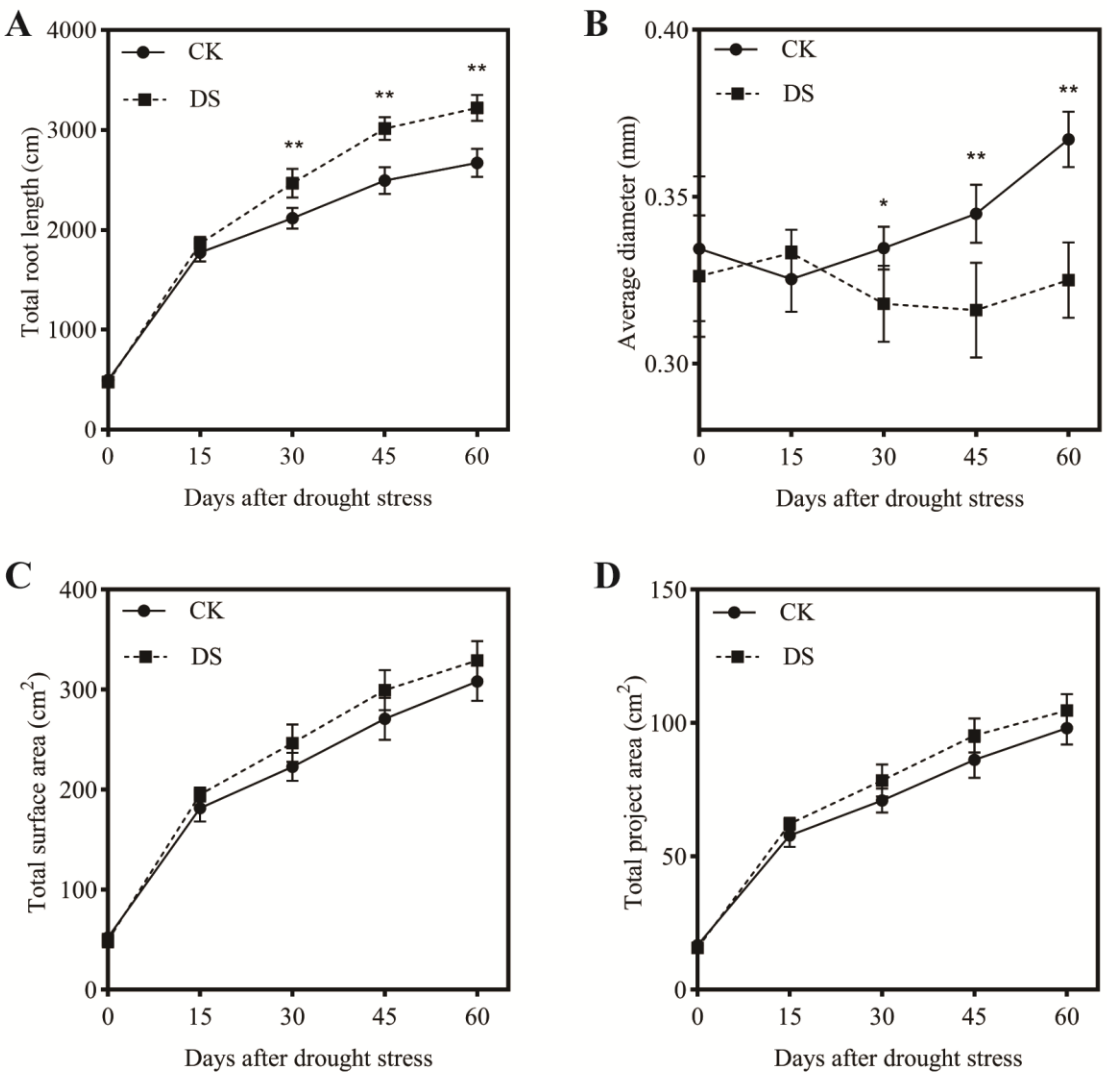

Additional file 2: Fig. S2. **Root morphological responses to drought.** Changes of total root length (A), total average diameter (B), total surface area (C), and total project area (D) of cotton roots during drought stress. Each data point represents the mean of five independent biological replicates (mean± SD). *Represents a statistically significant difference when compared with the control ($^{*}P$<0.05; $^{**}P$<0.01).
