# Supplementary material for: Tandem mass tag-based (TMT) quantitative proteomics analysis reveals the response of fine roots to drought stress in cotton (Gossypium hirsutum L.)
Source: BMC Plant Biol. 2020 Jul 11;20:328. doi: 10.1186/s12870-020-02531-z (PMC7353779; doi:10.1186/s12870-020-02531-z)
Supplement: Supplementary file 3 — Additional file 3: Figure S3. Length distribution of peptides identified by mass spectrometry (A); distribution of protein quantitative relative standard deviation (RSD) among repeated samples (B). [file 12870_2020_2531_MOESM3_ESM.pdf]

Fig. S3

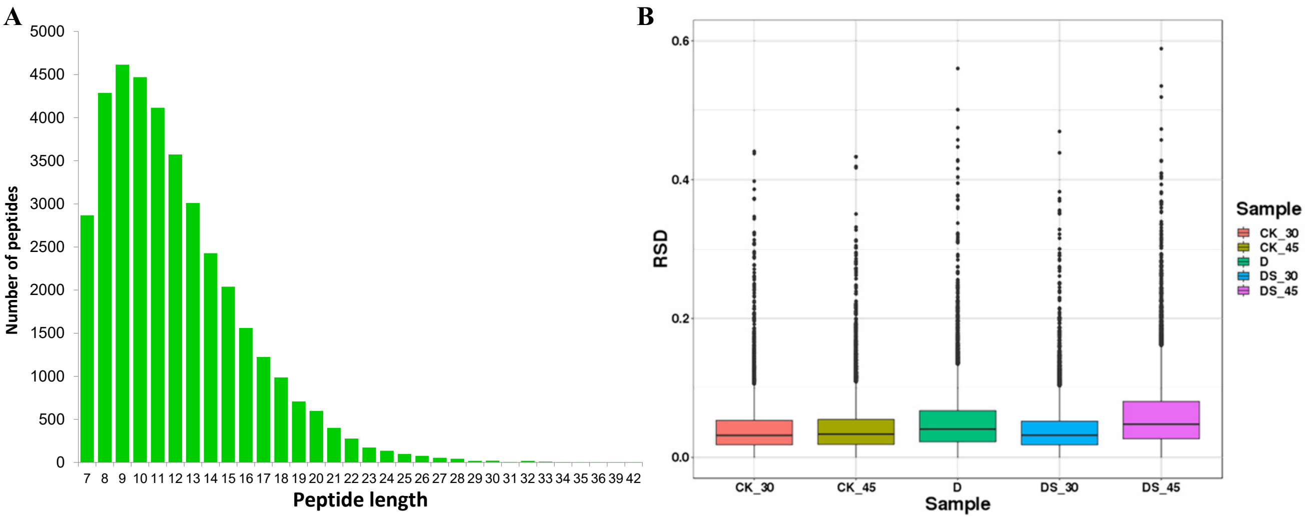

Additional file 3: Fig. S3. Length distribution of peptides identified by mass spectrometry (A); distribution of protein quantitative relative standard deviation (RSD) among repeated samples (B).
