# Supplementary material for: Tandem mass tag-based (TMT) quantitative proteomics analysis reveals the response of fine roots to drought stress in cotton (Gossypium hirsutum L.)
Source: BMC Plant Biol. 2020 Jul 11;20:328. doi: 10.1186/s12870-020-02531-z (PMC7353779; doi:10.1186/s12870-020-02531-z)
Supplement: Supplementary file 7 — Additional file 7: Figure S4. Gene ontology (GO) enrichment analysis of the differentially expressed proteins (DEPs) in “DS30 vs CK30” comparsion group. (A) Up-regulated DEPs and (B) down-regualted DEPs. [file 12870_2020_2531_MOESM7_ESM.pdf]

Fig. S4

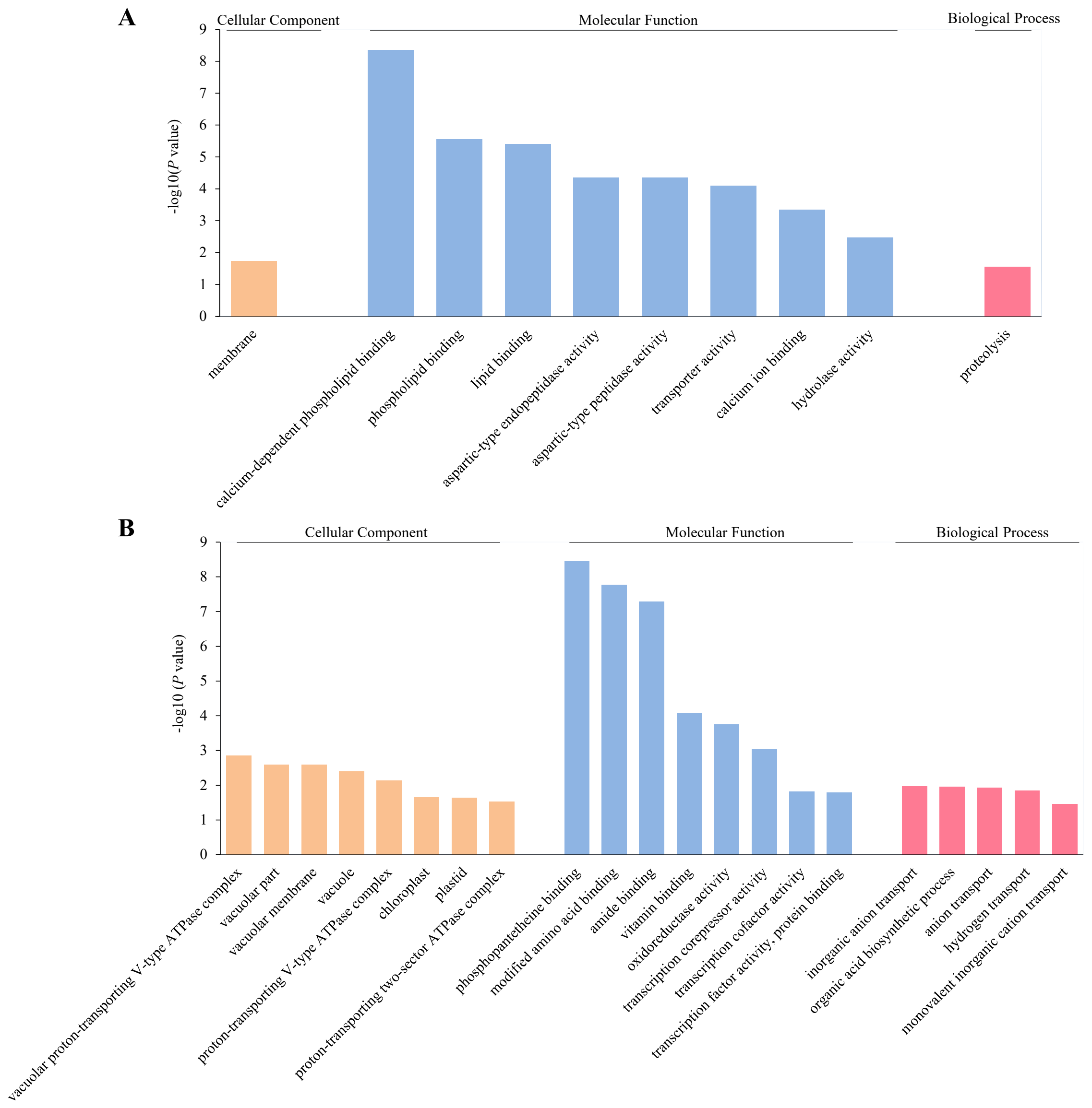

Additional file 7: Fig. S4. Gene ontology (GO) enrichment analysis of the up-regulated (A) and down-regulated (B) differentially expressed proteins (DEPs) in "DS30 vs CK30" comparsion group.
