# Supplementary material for: Tandem mass tag-based (TMT) quantitative proteomics analysis reveals the response of fine roots to drought stress in cotton (Gossypium hirsutum L.)
Source: BMC Plant Biol. 2020 Jul 11;20:328. doi: 10.1186/s12870-020-02531-z (PMC7353779; doi:10.1186/s12870-020-02531-z)
Supplement: Supplementary file 10 — Additional file 10:. Distributions of peptide fragment ion peak area. [file 12870_2020_2531_MOESM10_ESM.docx]

PRM is quantified using peak area. The fragment ion peak area distribution of the selected peptide in 15 samples is shown below:
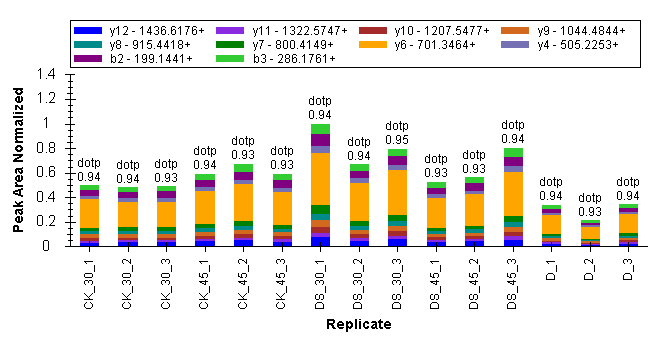


**Fig.1** **VVSINDYEDVPVNDEK fragment ion peak area distribution map.** Fragment ion peak area distribution of peptide VVSINDYEDVPVNDEK (corresponding to protein A0A1U8L2Y6) in 15 samples.


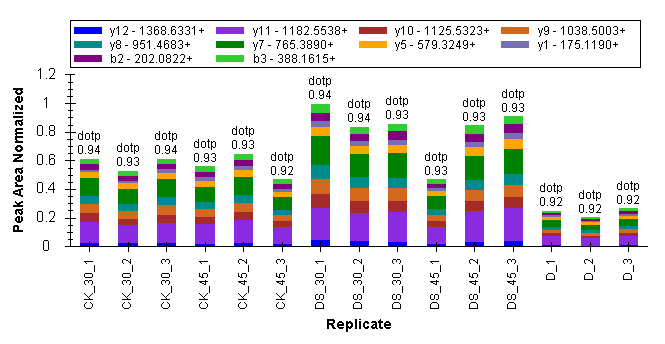


**Fig.2** **NSWGSSWGEAGYLR fragment ion peak area distribution map.** Fragment ion peak area distribution of peptide NSWGSSWGEAGYLR (A0A1U8L2Y6) in 15 samples.


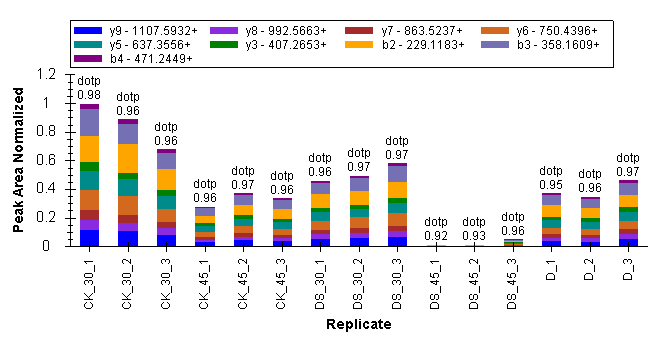


**Fig.3** **IDELITEFLK fragment ion peak area distribution map.** Fragment ion peak area distribution of peptide IDELITEFLK (A0A1U8P5X3) in 15 samples.


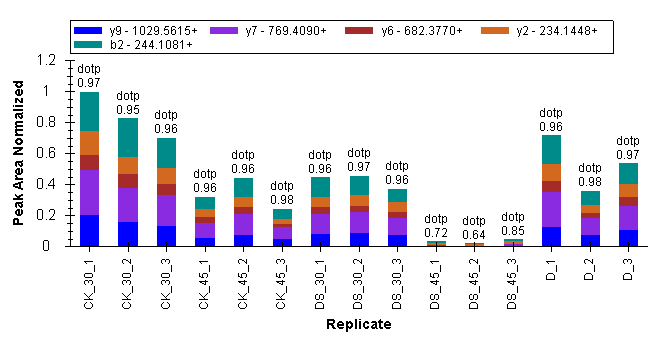


**Fig.4** **GWPSFLSAYTISK fragment ion peak area distribution map.** Fragment ion peak area distribution of peptide GWPSFLSAYTISK (A0A1U8P5X3) in 15 samples.


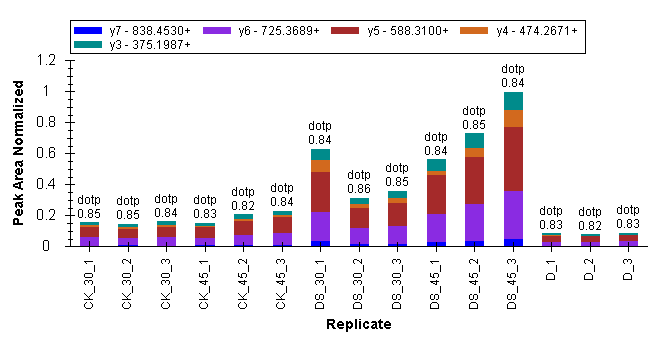


**Fig. 5** **LLNLHNVAER fragment ion peak area distribution map.** Fragment ion peak area distribution of peptide LLNLHNVAER (A0A0U2L631) in 15 samples.


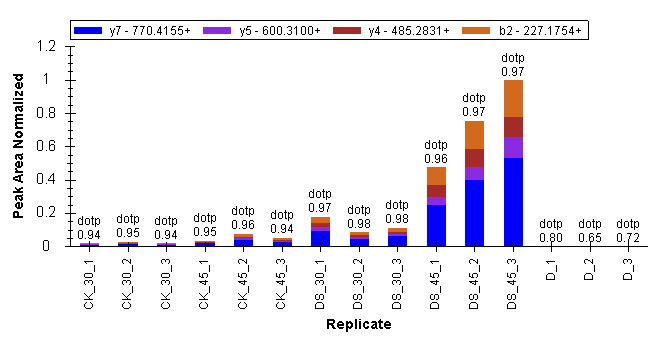


**Fig. 6 IIAVDVNPR fragment ion peak area distribution map.** Fragment ion peak area distribution of peptide IIAVDVNPR (A4L9H3) in 15 samples.


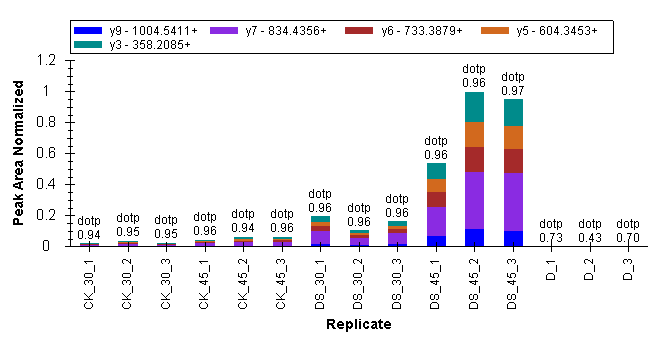


**Fig. 7 FGITEFVNPK fragment ion peak area distribution map.** Fragment ion peak area distribution of peptide FGITEFVNPK (A4L9H3) in 15 samples.


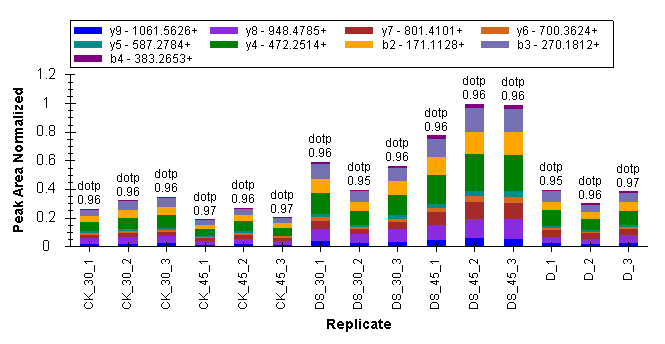


**Fig. 8** **AVVLFTLDPAER fragment ion peak area distribution map.** Fragment ion peak area distribution of peptide AVVLFTLDPAER (A0A1U8IN71) in 15 samples.


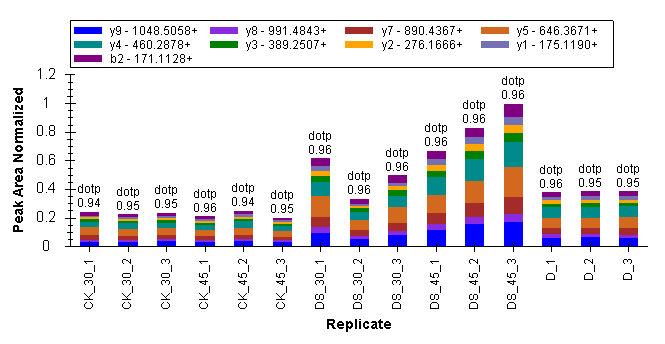


**Fig. 9** **LGTDEWALTR fragment ion peak area distribution map.** Fragment ion peak area distribution of peptide LGTDEWALTR (A0A1U8IN71) in 15 samples.


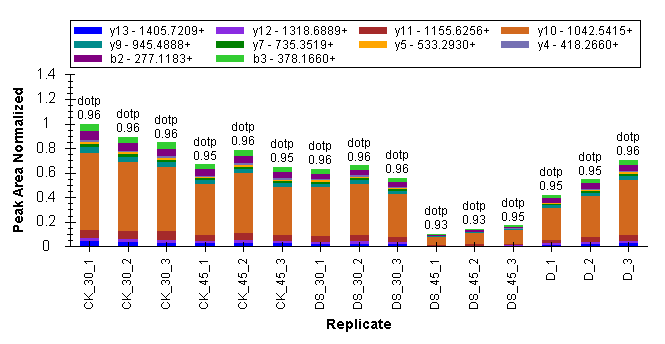


**Fig. 10** **FETLSYLPPLSDDSIAK fragment ion peak area distribution map.** Fragment ion peak area distribution of peptide FETLSYLPPLSDDSIAK (A0A1U8PZJ8) in 15 samples.


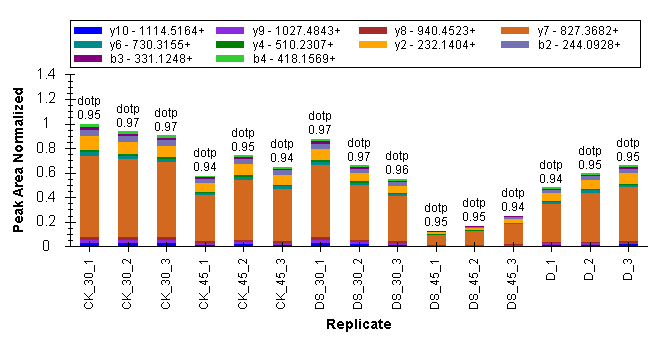


**Fig. 11 ENSSIPGYYDGR fragment ion peak area distribution map.** Fragment ion peak area distribution of peptide ENSSIPGYYDGR (A0A1U8PZJ8) in 15 samples.


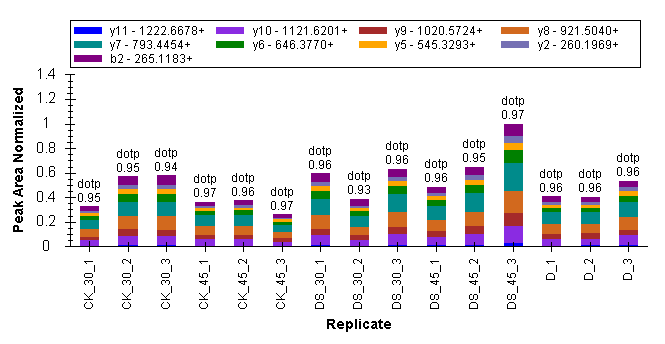


**Fig. 12 YTTVQFTGEVLK fragment ion peak area distribution map.** Fragment ion peak area distribution of peptide YTTVQFTGEVLK (A0A1U8L4B5) in 15 samples.


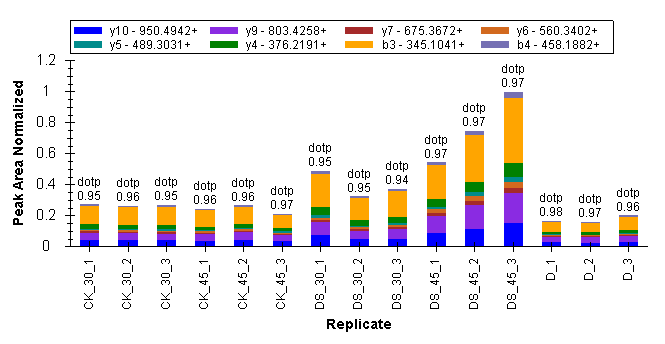


**Fig. 13 DNDLFGADAIGTAK fragment ion peak area distribution map.** Fragment ion peak area distribution of peptide DNDLFGADAIGTAK (A0A1U8NBT7) in 15 samples.


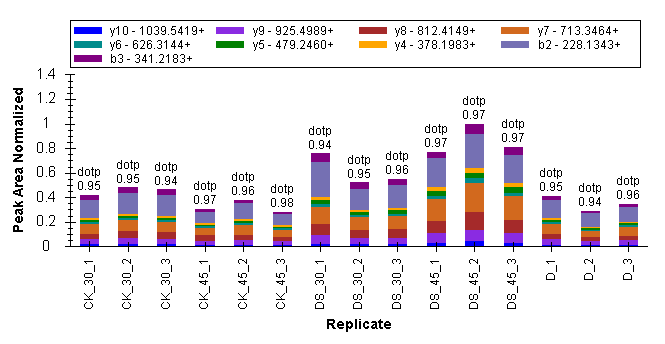


**Fig. 14 INLVSFTGSSK fragment ion peak area distribution map.** Fragment ion peak area distribution of peptide INLVSFTGSSK (A0A1U8IUK2) in 15 samples.


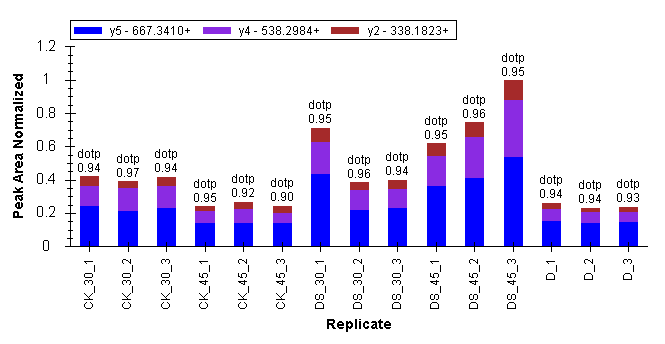


**Fig. 15 LLLHESIYR fragment ion peak area distribution map.** Fragment ion peak area distribution of peptide LLLHESIYR (A0A1U8IUK2) in 15 samples.


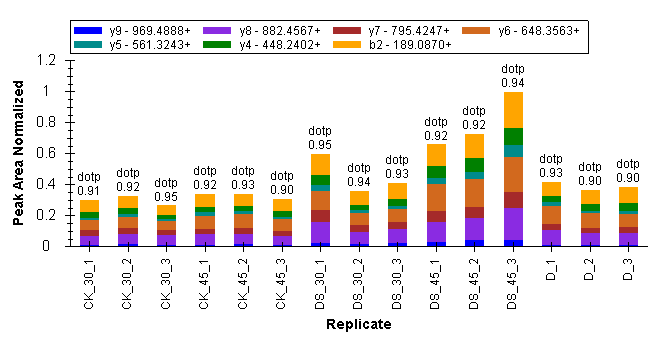


**Fig. 16 TSSFSLTEAK fragment ion peak area distribution map.** Fragment ion peak area distribution of peptide TSSFSLTEAK (A0A1U8PJA6) in 15 samples.


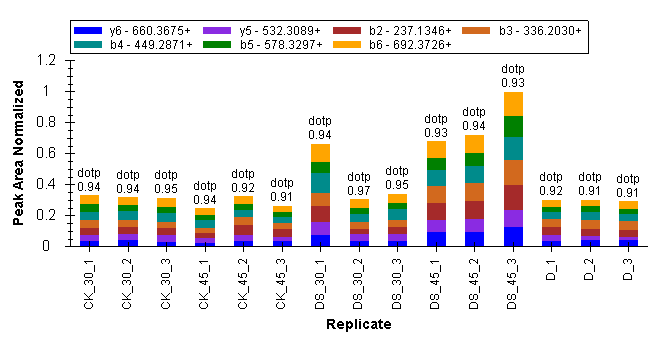


**Fig. 17 HVVLENVQNASLK fragment ion peak area distribution map.** Fragment ion peak area distribution of peptide HVVLENVQNASLK (A0A1U8PJA6) in 15 samples.


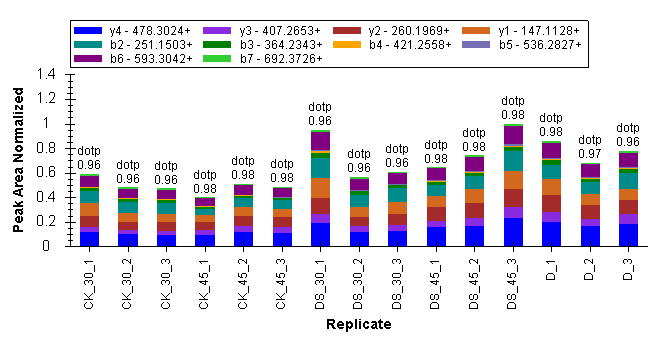


**Fig. 18 LHIGDGVAFLK fragment ion peak area distribution map.** Fragment ion peak area distribution of peptide LHIGDGVAFLK (A0A1U8JR63) in 15 samples.


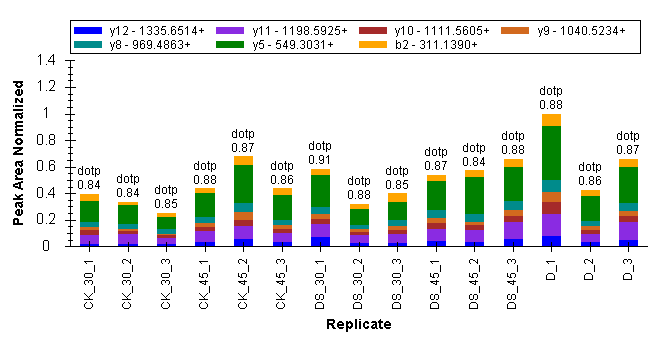


**Fig. 19 FYNSEIHSAAFCLPSFAK fragment ion peak area distribution map.** Fragment ion peak area distribution of peptide FYNSEIHSAAFCLPSFAK (A0A1U8JR63) in 15 samples.


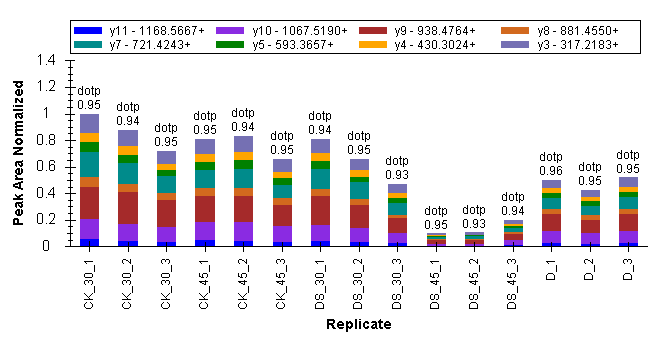


**Fig. 20 VTEGCGAYIAVK fragment ion peak area distribution map.** Fragment ion peak area distribution of peptide VTEGCGAYIAVK (A0A1U8JXE9) in 15 samples.


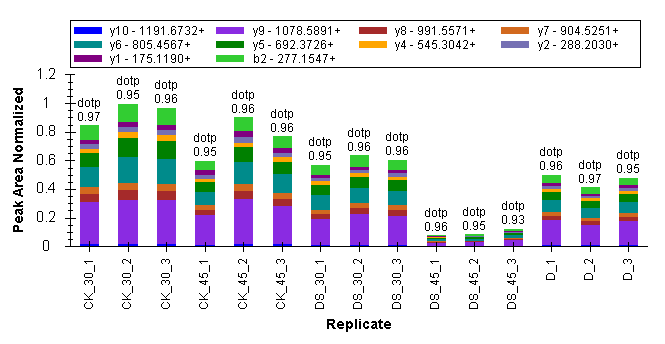


**Fig. 21 YLSSVLFQELR fragment ion peak area distribution map.** Fragment ion peak area distribution of peptide YLSSVLFQELR (A0A1U8JXE9) in 15 samples.


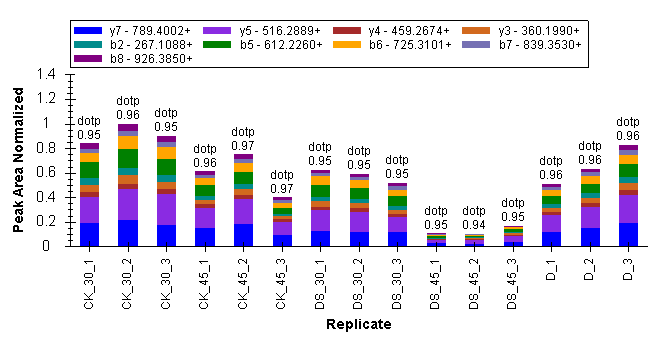


**Fig. 22 HETEDINSFSWGVANR fragment ion peak area distribution map.** Fragment ion peak area distribution of peptide HETEDINSFSWGVANR (A0A1U8KSQ2) in 15 samples.


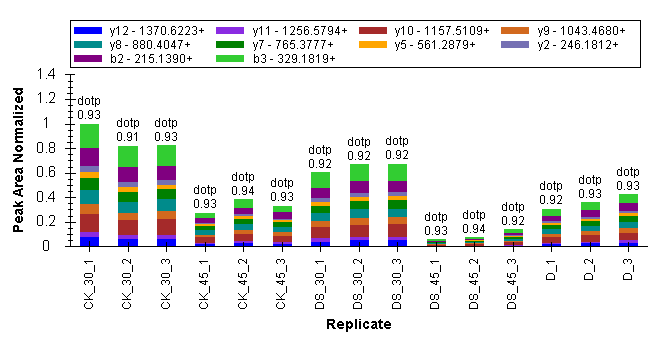


**Fig. 23 TINVNYDGFAEDVK fragment ion peak area distribution map.** Fragment ion peak area distribution of peptide TINVNYDGFAEDVK (A0A1U8LN42) in 15 samples.


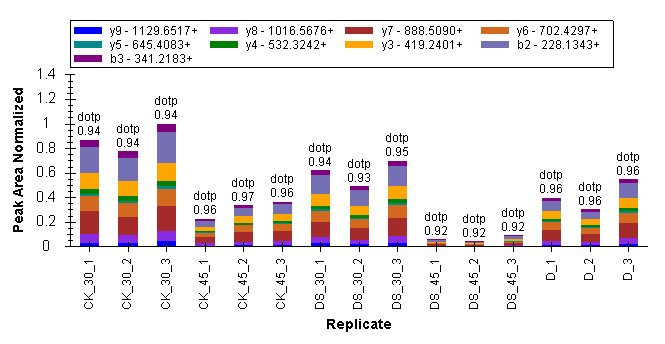


**Fig. 24 LNLQWGLIPFR fragment ion peak area distribution map.** Fragment ion peak area distribution of peptide LNLQWGLIPFR (A0A1U8LN42) in 15 samples.


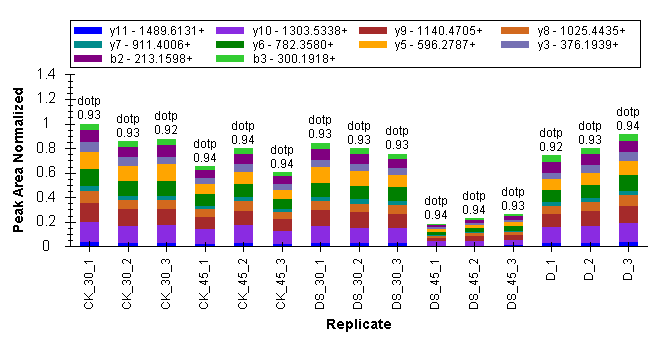


**Fig. 25 LVSWYDNEWGYSNR fragment ion peak area distribution map.** Fragment ion peak area distribution of peptide LVSWYDNEWGYSNR (A0A1U8PS93) in 15 samples.


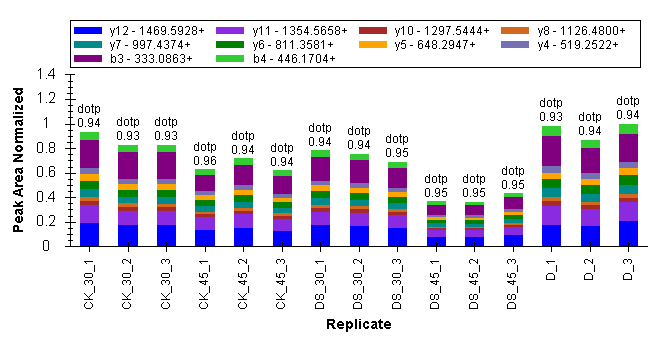


**Fig. 26 GDCIIDGGNEWYENTER fragment ion peak area distribution map.** Fragment ion peak area distribution of peptide GDCIIDGGNEWYENTER (A0A1U8NRN0) in 15 samples.


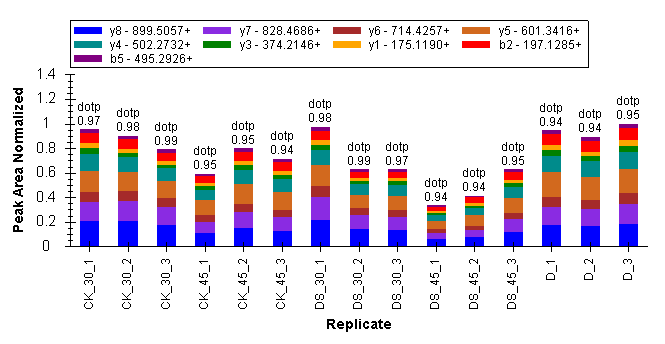


**Fig. 27 VPANLVQAQR fragment ion peak area distribution map.** Fragment ion peak area distribution of peptide VPANLVQAQR (A0A1U8NRN0) in 15 samples.


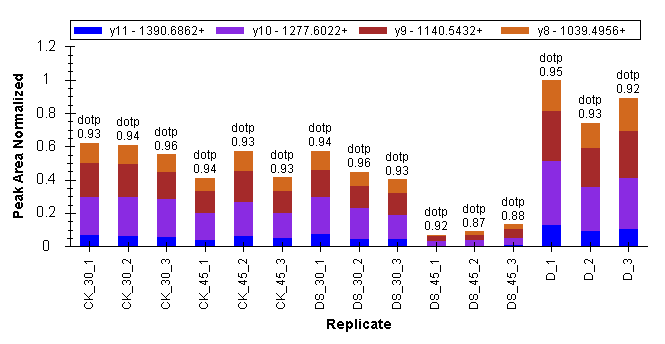


**Fig. 28 YANSNIQIHTFNQSQYPR fragment ion peak area distribution map.** Fragment ion peak area distribution of peptide YANSNIQIHTFNQSQYPR (D2D2Z0) in 15 samples.


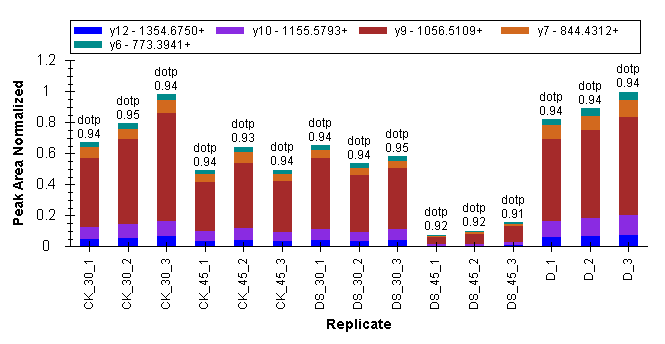


**Fig. 29 VQLLEIAQVPDAHVNEFK fragment ion peak area distribution map.** Fragment ion peak area distribution of peptide VQLLEIAQVPDAHVNEFK (D2D2Z0) in 15 samples.
