# Supplementary material for: Tandem mass tag-based (TMT) quantitative proteomics analysis reveals the response of fine roots to drought stress in cotton (Gossypium hirsutum L.)
Source: BMC Plant Biol. 2020 Jul 11;20:328. doi: 10.1186/s12870-020-02531-z (PMC7353779; doi:10.1186/s12870-020-02531-z)
Supplement: Supplementary file 15 — Additional file 15: Method S1. Parallel reaction monitoring (PRM) analysis procedure [file 12870_2020_2531_MOESM15_ESM.docx]

**S1. Experimental Procedures**

**S1.1 Protein Extraction and Trypsin Digestion**

The same as TMT test.

**S1.2 LC-MS/MS Analysis**

The tryptic peptides were dissolved in 0.1% formic acid (solvent A), directly loaded onto a home-made reversed-phase analytical column. The gradient was comprised of an increase from 6% to 25% solvent B (0.1% formic acid in 98% acetonitrile) over 40 min, 25% to 35% in 12 min and climbing to 80% in 4 min then holding at 80% for the last 4 min, all at a constant flow rate of 500 nL/min on an EASY-nLC 1000 UPLC system.

The peptides were subjected to NSI source followed by tandem mass spectrometry (MS/MS) in Q ExactiveTM Plus (Thermo) coupled online to the UPLC. The electrospray voltage applied was 2.0 kV. The m/z scan range was 380 to 1180 for full scan, and intact peptides were detected in the Orbitrap at a resolution of 70,000. Peptides were then selected for MS/MS using NCE setting as 27 and the fragments were detected in the Orbitrap at a resolution of 17,500. A data-independent procedure that alternated between one MS scan followed by 20 MS/MS scans. Automatic gain control (AGC) was set at 3E6 for full MS and 1E5 for MS/MS. The maxumum IT was set at 50 ms for full MS and 150 ms for MS/MS. The isolation window for MS/MS was set at 1.6 m/z.

**S1.3 Data Analysis**

The resulting MS data were processed using Skyline (v.3.6). Peptide settings: enzyme was set as Trypsin [KR/P], Max missed cleavage set as 0. The peptide length was set as 7-25, Variable modification was set as Carbamidomethyl on Cys and oxidation on Met, and max variable modifications was set as 3. Transition settings: precursor charges were set as 2, 3, ion charges were set as 1, 2, ion types were set as b, y, p. The product ions were set as from ion 3 to last ion, the ion match tolerance was set as 0.02 Da.
